# Off-the-shelf invariant NKT cells expressing anti-PSCA CAR and IL-15 promote pancreatic cancer regression in mice

**DOI:** 10.1172/JCI179014

**Published:** 2025-04-15

**Authors:** Zhenyu Dai, Zheng Zhu, Zhiyao Li, Lei Tian, Kun-Yu Teng, Hanyu Chen, Li-Shu Wang, Jianying Zhang, Laleh Melstrom, Michael A. Caligiuri, Jianhua Yu

**Affiliations:** 1 Department of Hematology and Hematopoietic Cell Transplantation, City of Hope National Medical Center, Los Angeles, California, USA.; 2 Division of Hematology/Oncology, Department of Medicine, School of Medicine, and; 3 The Clemons Family Center for Transformative Cancer Research, Chao Family Comprehensive Cancer Center, University of California, Irvine, California, USA.; 4 Department of Computational and Quantitative Medicine,; 5 Division of Surgical Oncology, Department of Surgery, and; 6 Hematologic Malignancies Research Institute, City of Hope National Medical Center, Los Angeles, California, USA.; 7 City of Hope Comprehensive Cancer Center, Los Angeles, California, USA.

**Keywords:** Immunology, Oncology, Immunotherapy, NKT cells

## Abstract

Pancreatic ductal adenocarcinoma cancer (PDAC) continues to pose a significant health burden, with a 5-year survival rate of only 10%. Prostate stem cell antigen (PSCA) is highly expressed on the surface of tumor cells of most PDAC patients, with minimum expression in most normal tissues. Here, we generated cryopreserved, off-the-shelf, allogeneic PSCA chimeric antigen receptor (CAR) invariant NKT (iNKT) cells using human peripheral blood mononuclear cells as a cell source. In multiple in vitro and in vivo PDAC models, freshly manufactured PSCA CAR_sIL-15 iNKT cells and frozen-thawed, off-the-shelf PSCA CAR_sIL-15 iNKT cells demonstrate comparable efficacies, and both show remarkable suppression of PSCA-positive and gemcitabine-resistant PDAC. Importantly, off-the-shelf cryopreserved PSCA CAR_sIL-15 iNKT cells show equivalent efficacy when compared with PSCA CAR T cells using the same PSCA CAR and in the same PDAC model; however, PSCA CAR_sIL-15 iNKT cells do not appear to induce systemic toxicity or graft-versus-host disease, thus allowing for multiple infusions to control recurrent disease. Collectively, our study suggests that PSCA CAR_sIL-15 iNKT cells merit clinical investigation for PDAC patients exhibiting positive PSCA expression. The therapy could be given as a single agent or in combination with established therapeutic modalities for PDAC.

## Abstract 2

<h2>Readily available “off-the-shelf” PSCA CAR_sIL15 NKT cells show excellent anti-PDAC activities without causing GvHD in vitro and in vivo.</h2>

## Introduction

Pancreatic ductal adenocarcinoma cancer (PDAC) accounts for more than 90% of pancreatic cancer cases and is one of the most aggressive solid tumors, characterized by a high rate of morbidity and mortality. It accounts for 7% of all cancer-related deaths, and the general 5-year survival rate for PDAC patients is just 10% (1). These alarming statistics raise concerns, as PDAC is predicted to become the second leading cause of cancer-related deaths by 2030 (2). The poor prognosis of PDAC can be attributed to several factors, including the absence of specific symptoms, leading to diagnosis at advanced stages with local and/or distant metastases. Additionally, PDAC cells exhibit a high resistance to standard chemotherapy, and the tumor microenvironment (TME) is characterized by highly immunosuppressive and metabolic challenges. To date, salvage chemotherapy regimens remain the primary option for treating advanced PDAC. Gemcitabine (2’,2’-difluorodeoxycytidine), a nucleoside analog, is the first-line intervention to treat advanced PDAC. However, despite its use, overall survival rates remain unsatisfactory, leaving few alternatives for patients who have failed gemcitabine-based therapy (3, 4). Therefore, it is imperative to prioritize the development of innovative and effective therapies to fight PDAC.

In recent years, substantial progress has been made with chimeric antigen receptor T (CAR-T) cell therapies, primarily for the treatment of lymphoid malignancies. These therapies have shown promise by inducing remissions and improving long-term relapse-free survival in B cell leukemia, lymphoma, and multiple myeloma. Unfortunately, the results of clinical trials indicate that CAR-T cell therapy has had limited success in treating solid tumors, including PDAC (5, 6). Several barriers must be overcome, such as the challenge of limited CAR-T cell infiltration at tumor sites and the immunosuppressive effects of the TME. These effects lead to impaired CAR-T cell proliferation, onset of exhaustion, and thus reduced efficacy. In addition, inserting CAR genes into polyclonal activated T lymphocytes results in cell products with high functional heterogeneity, which may compromise their antitumor potential and increase the risk of toxicity, including cytokine storm (7). Furthermore, the current application of CAR-T cells is primarily autologous, aimed at avoiding graft-versus-host disease (GvHD). This limits recurrent administration and widespread distribution while incurring high costs. The development of allogeneic, off-the-shelf CAR-T cell therapies is still in progress. Studies are ongoing to explore the potential of specific lymphocyte subsets, such as NK cells, γ δT, IL-9–secreting T cells, or NKT cells, which have been reported to be superior in terms of cell-mediated cytotoxicity, tumor infiltration, or desired cytokine production. These investigations represent a promising avenue for CAR-based immunotherapy (7–9).

Type I NKT cells (or invariant NKT [iNKT] cells) are an evolutionarily conserved sublineage of T cells that express the invariant TCR-α chain (Vα24-Jα18) (10). They possess unique characteristics that are intermediate between NK and T cells and exhibit an ability to recognize self- and microbial-derived glycolipids presented by the monomorphic human leukocyte antigen (HLA) class I–like molecule CD1d (10). Unlike HLA molecules, which exhibit genetic polymorphism and ubiquitous expression, CD1d gene expression is monomorphic and limited to specific cell types. This unique characteristic minimizes the potential for autologous or allogeneic iNKT cells to cause toxicity, regardless of HLA allele expression (11). iNKT cells offer distinct mechanistic advantages over bulk T cell populations when applied to CAR-based immunotherapy. They exhibit the capacity to traffic to solid tumors in response to chemokines produced by tumor cells, stromal cells, and tumor-associated macrophages (TAM) (12). This migration of iNKT cells into primary tumors correlates with better outcomes in various types of tumors (12, 13).

Several studies have demonstrated that donor-derived iNKT cells can effectively inhibit GvHD while preserving their antitumor activity (14, 15). In pediatric leukemia patients who received haploidentical transplants, the reconstitution of iNKT cells in peripheral blood has been associated with long-term remission (16). Furthermore, during the preparation of our manuscript, it was reported that autologous CAR-NKT cells have superior antitumor activity compared with CAR-T cells (17). Given these promising features, here we explore the potential of using iNKT cells for CAR modification in the treatment of PDAC.

Targeting the right antigen is critical to ensure the safety and effectiveness of CAR-based therapy. Prostate stem cell antigen (PSCA) is a glycosylphosphatidylinositol-linked cell-surface antigen that plays a critical role in promoting cell-cycle progression and boosting tumor cell proliferation. Moreover, the presence of metastasis and advanced clinical stages in prostate cancers is directly linked to the levels of PSCA protein (18, 19). Also, the overexpression of PSCA in PDAC begins during the early stages of malignant transformation and can be detected in 60%–80% of patients diagnosed with PDAC. In contrast, PDAC expression in normal pancreatic tissue is very low, providing a strong rationale for PSCA-targeting immunotherapy (20, 21). Previously, we reported that PSCA CAR NK cells expressing soluble IL-15 (sIL-15) showed significant and specific tumor-suppressive effects on PSCA^+^ PDAC, both in vitro and in vivo (22). These results underscore the promising potential of targeting PSCA to treat PDAC.

The objective of this study was to develop off-the-shelf human PSCA CAR iNKT cells expressing sIL-15 to enhance the antitumor functions of iNKT cells without inducing toxicity for sustained control of PDAC tumors. In addition, Liu et al. demonstrated that IL-15–expressing iNKT cells are not subjected to TAM inhibition or hypoxia, thereby significantly increasing their antimetastatic activity (23). Therefore, sIL-15 was also incorporated into our CAR construct (PSCA CAR_sIL-15). Our results demonstrate that PSCA CAR_sIL-15 iNKT cells exhibit enduring antitumor efficacy in vitro and in vivo without causing notable toxicity in multiple models, including the orthotopic PDAC model and the metastatic PDAC model. Moreover, we observed that PSCA CAR_sIL-15 iNKT cells efficiently kill PDAC cells that had developed resistance to first-line standard chemotherapy with gemcitabine in vitro and in vivo. Off-the-shelf PSCA CAR_sIL-15 iNKT cells were further validated to have comparable antitumor capabilities even after undergoing a freeze-thaw cycle without risk of GvHD when compared with PSCA CAR_sIL-15 T cells generated from the same donor and to fresh PSCA CAR_sIL-15 iNKT cells. These preclinical evaluations provide a robust foundation for exploring the clinical applications of allogeneic off-the-shelf PSCA CAR_sIL-15 iNKT cells.

## Results

### PSCA CAR_sIL-15 iNKT cells demonstrate excellent in vitro expansion and have low expression of exhaustion markers.

**Figure 1:**
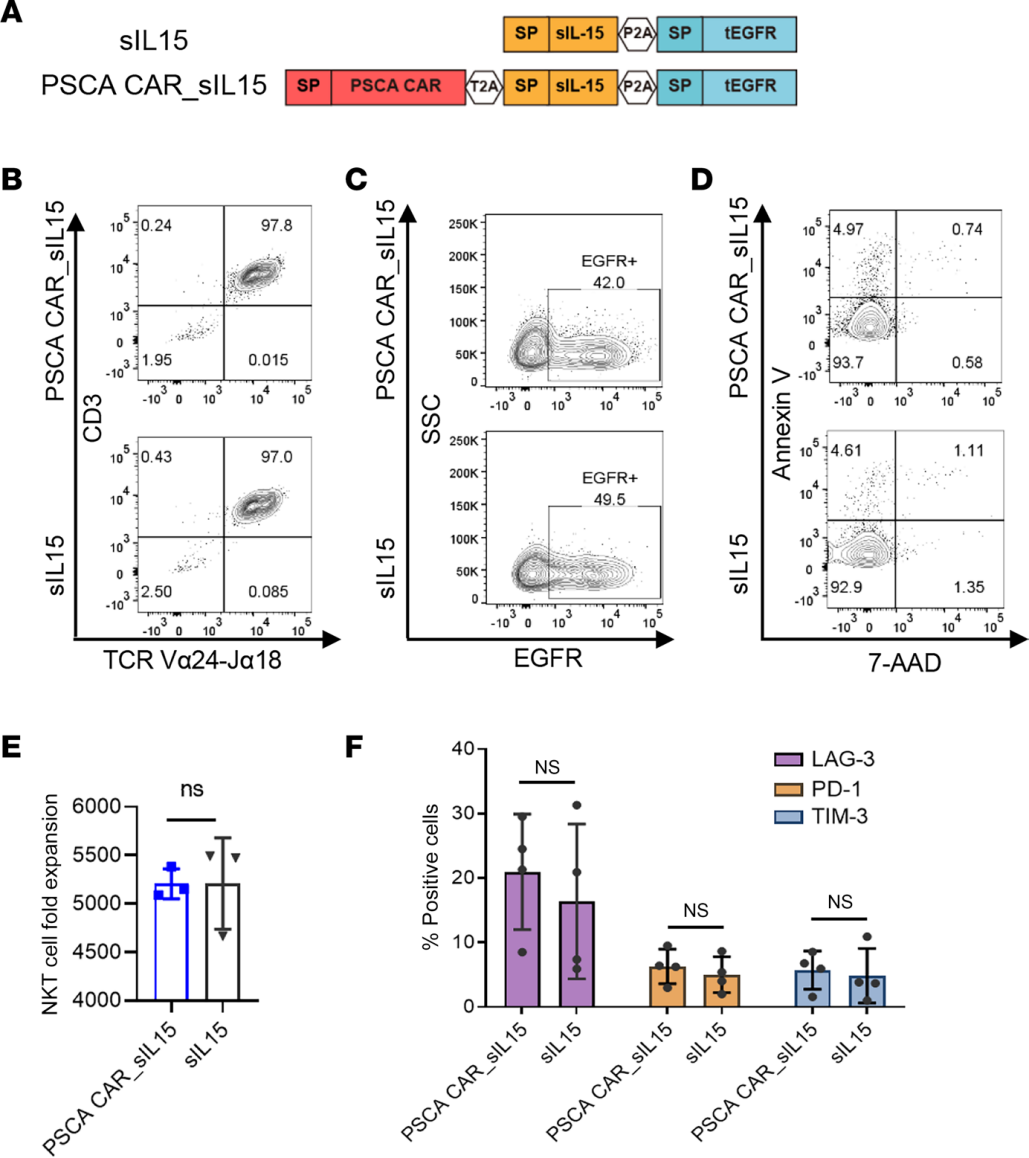
iNKT cells expressing the PSCA CAR_sIL-15 construct demonstrate excellent in vitro expansion and show low surface expression of exhaustion markers. (**A**) Schematic diagrams of the clinical grade vectors. tEGFR was included as both a detection marker and a safety switch, allowing for in vivo iNKT cell depletion by administering an anti-EGFR antibody. (**B**) Representative flow cytometric analysis of PSCA CAR_sIL-15 iNKT cells and sIL-15 iNKT cells shows the proportion of CD3 and iNKT (TCR Vα24-Jα18) expression 2 days after transduction. sIL-15 iNKT cells and PSCA CAR_sIL-15 iNKT cells show high NKT purity of approximately 97%. The experiment was conducted with 3 donors with similar results. (**C**) The transduction ratio of PSCA CAR_sIL-15/sIL-15 iNKT cells was detected by measuring tEGFR expression 2 days after transduction and analyzed by flow cytometry. The transduction efficiencies, approximately 42%, were similar in both sIL-15 iNKT cells and PSCA CAR_sIL-15 iNKT cells. The experiment was conducted with 3 donors with similar results. SSC, side scatter. (**D**) The level of apoptosis of sIL-15 iNKT cells and PSCA CAR_sIL-15 iNKT cells was measured by the coexpression of annexin V and 7-AAD 2 days after transduction by flow cytometry. Both sIL-15 iNKT cells and PSCA CAR_sIL-15 iNKT cells exhibited very low levels of apoptosis. (**E**) Quantification of PSCA CAR_sIL-15 and sIL-15 iNKT cell fold expansion following 12 days of secondary expansion (mean ± SD, *n* = 3). Not significant (Student’s *t* test). Both sIL-15 iNKT cells and PSCA CAR_sIL-15 iNKT cells can be expanded more than 5,000-fold. (**F**) Surface expression of exhaustion markers LAG-3, PD-1, and TIM-3 on sIL-15 iNKT cells and PSCA CAR_sIL-15 iNKT cells, measured by flow cytometry. The results are displayed as mean ± SD (*n* = 3). Not significant (2-way ANOVA).

Human primary iNKT cells isolated and expanded from human PBMCs were engineered to express soluble IL-15 alone (sIL-15 iNKT) or both PSCA CAR and sIL-15 (PSCA CAR_sIL-15 iNKT; Figure 1A). Our constructs also included a truncated EGFR (tEGFR) as a marker to detect successful transduction and as a safety switch, the latter of which allows for in vivo depletion of PSCA CAR_sIL-15 iNKT cells by administering a clinical-grade anti-EGFR antibody, cetuximab (22). The sIL-15 iNKT cells and PSCA CAR_sIL-15 iNKT cells showed high (97%) iNKT purity, identified by the marker of TCR Vα24-Jα18. Furthermore, the overall transduction efficiencies, around 42%, were similar for both sIL-15 iNKT cells and PSCA CAR_sIL-15 iNKT cells, as assessed by EGFR expression 3 days after the cells underwent transduction. We also detected the basal apoptosis levels of sIL-15 iNKT cells and PSCA CAR_sIL-15 iNKT cells, showing that both populations exhibited very low basal levels (~5%) of apoptosis (Figure 1, B–D). These data demonstrate our newly generated PSCA CAR_sIL-15 iNKT cells show high purity, have a high rate of CAR transduction, and are generally healthy several days after transduction. After in vitro culture with α-galactosylceramide (α-GalCer), both sIL-15 and PSCA CAR_sIL-15 iNKT cells can be expanded more than 5,000-fold (Figure 1E) and the expanded iNKT cells display low surface density expression of exhaustion markers LAG-3, PD-1, and TIM-3 (Figure 1F). These data demonstrate the successful engineering, manufacturing, and expansion of PSCA CAR_sIL-15 iNKT cells.

### PSCA CAR_sIL-15 iNKT cells exhibit potent antitumor activity against human PDAC cell lines in vitro.

**Figure 2:**
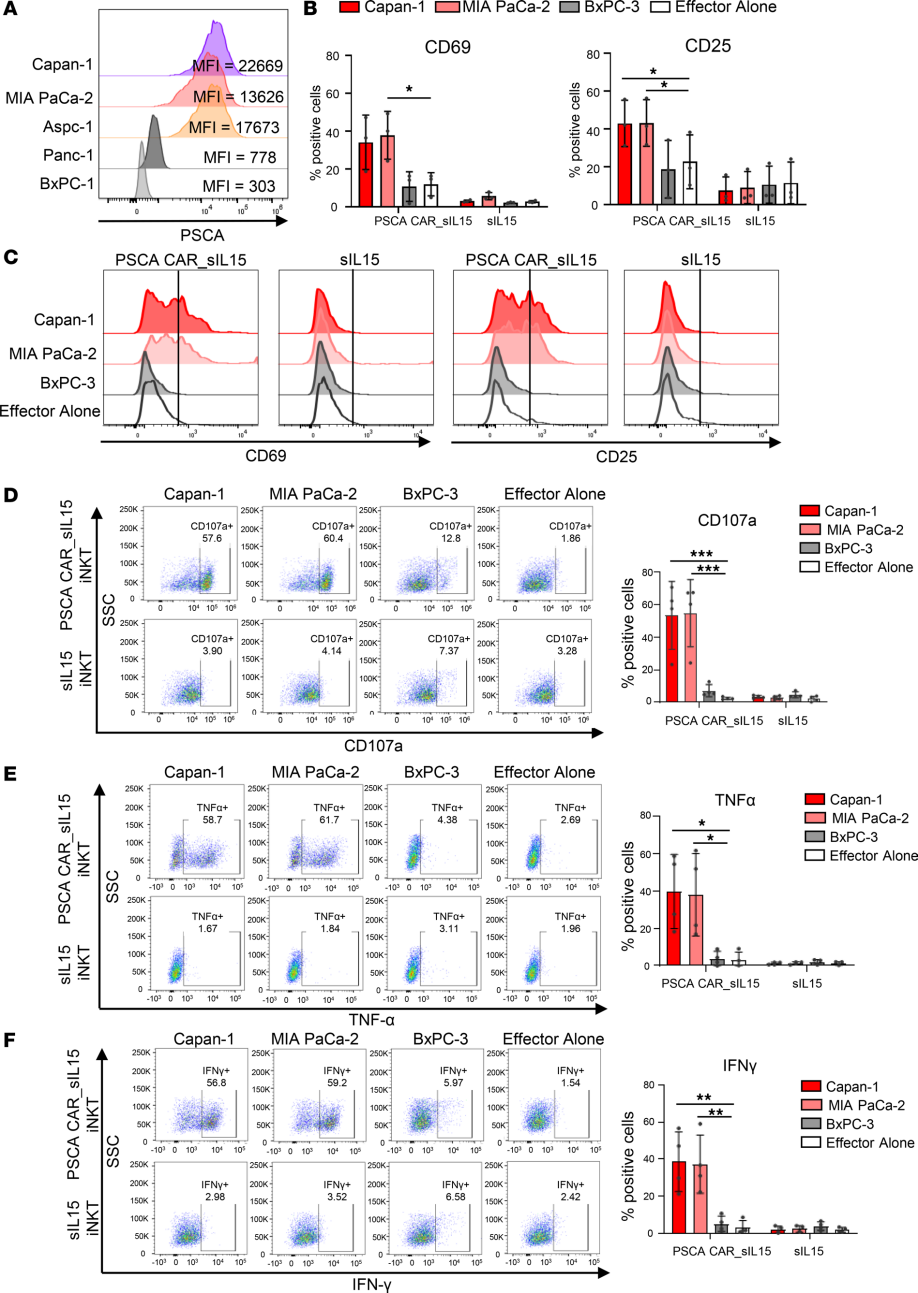
PSCA CAR_sIL-15 iNKT cells demonstrate PSCA^+^ PDAC cell-specific activation. (**A**) Surface density expression of PSCA on human PDAC cell lines was measured by mean fluorescent intensity (MFI) using flow cytometry. Capan-1, MIA PaCa-2, and Aspc-1 cells highly expressed PSCA, while Panc-1 and BxPC-3 cells had low PSCA expression. (**B**) Summary of percentages of sIL-15 iNKT cells and PSCA CAR_sIL-15 iNKT cells positive for CD69 and CD25 following a 24-hour coincubation with Capan-1, MIA PaCa-2, or BxPC-3 (gated on iNKT cells). Data are presented as mean ± SD (*n* = 3). (**C**) Representative flow cytometric analysis shows the expression of CD69 and CD25 on sIL-15 iNKT cells and PSCA CAR_sIL-15 iNKT cells after coincubation with target cells. (**D**) Representative flow cytometric analysis (left) and summary graph (right) show CD107a expression on sIL-15 iNKT cells and PSCA CAR_sIL-15 iNKT cells after coincubation with target cells. (**E**) Representative flow cytometric analysis (left) and summary graph (right) show TNF-α expression in sIL-15 iNKT cells and PSCA CAR_sIL-15 iNKT cells after coincubation with target cells. (**F**) Representative flow cytometric analysis (left) and summary graph (right) show IFN-γ expression of sIL-15 iNKT cells and PSCA CAR_sIL-15 iNKT cells after coincubation with target cells. All experiments were repeated using ≥ 3 donors and presented as mean ± SD (**B**, **D**, **E**, and **F**). Statistical analyses were performed using 1-way ANOVA, with *P* values corrected for multiple comparisons using the Holm-Šídák method.*P < 0.05; **P < 0.01; ***P < 0.001.

Previously, we reported that PSCA is highly expressed in primary PDAC tumor samples, and this expression was correlated with a poor prognosis for patients (22). In our subsequent functional validation to assess the antitumor activity of PSCA CAR_sIL-15 iNKT cells, we first measured the expression levels of PSCA in 5 different human PDAC cell lines using flow cytometry. We found that Capan-1, MIA PaCa-2, and Aspc-1 cells highly expressed PSCA, while Panc-1 and BxPC-3 cells had low PSCA expression (Figure 2A). To evaluate the anti-PDAC capability of PSCA CAR_sIL-15 iNKT cells in vitro, PSCA CAR_sIL-15 iNKT cells or sIL-15 iNKT cells were cocultured with different PDAC cell lines at an effector: target (E:T) ratio of 1:1 for 6 hours. Compared with PSCA CAR_sIL-15 iNKT cells alone, PSCA CAR_sIL-15 iNKT cells expressed higher levels of the iNKT cell-activation markers CD69 and CD25 after coculturing with PSCA^+^ Capan-1 cells and PSCA^+^ MIA PaCa-2 cells, but not when cocultured with PSCA^–^ BxPC-3 cells (Figure 2, B and C). In contrast, sIL-15 iNKT cells did not exhibit this phenotype (Figure 2, B and C).

Degranulation is a prerequisite for immune cell perforin-mediated killing. PSCA CAR_sIL-15 iNKT cells upregulated expression of CD107a (a surrogate marker for degranulation) when cocultured with PSCA^+^ PDAC cells but not with PSCA^–^ PDAC cells. In contrast, PSCA^+^ PDAC cells and PSCA^–^ PDAC cells did not activate sIL-15 iNKT cells (Figure 2D). Moreover, PSCA CAR_sIL-15 iNKT cells produced more proinflammatory cytokines, TNF-α and IFN-γ, in response to PSCA^+^ cells, compared with PSCA^–^ PDAC cells and with sIL-15 iNKT cells (Figure 2, E and F, respectively).

**Figure 3:**
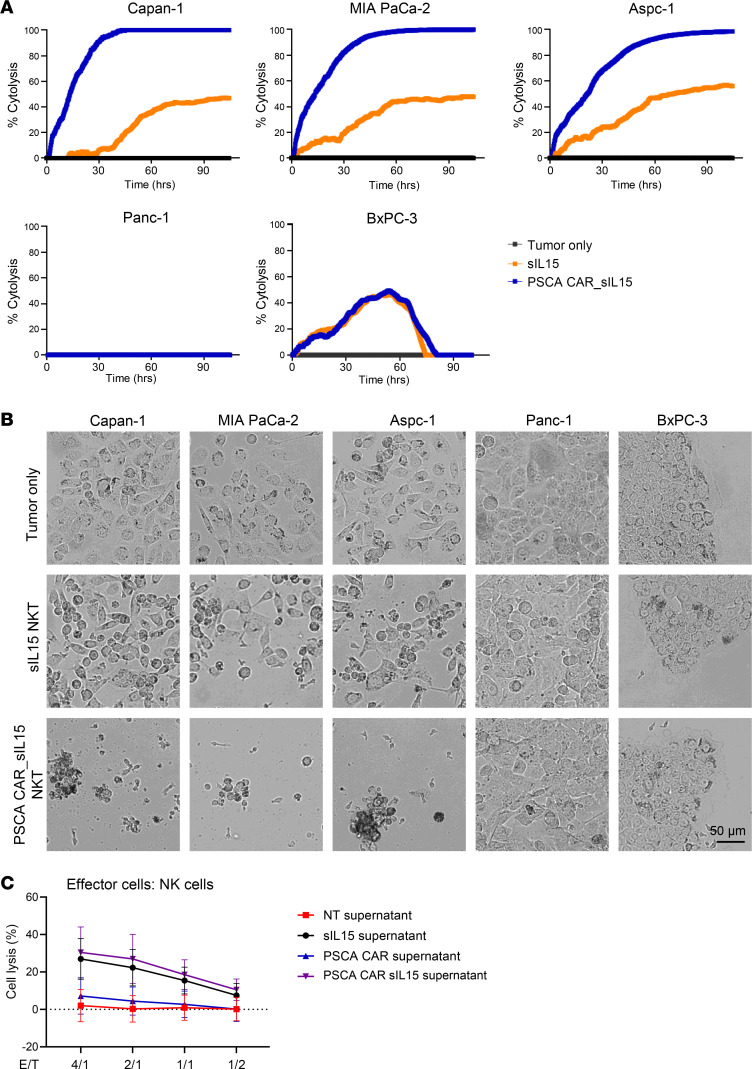
PSCA CAR_sIL-15 iNKT cells demonstrate potent and specific cytotoxicity against human PSCA^+^ PDAC cell lines in vitro. (**A**) RTCA results measuring cytotoxicity of sIL-15 iNKT cells and PSCA CAR_sIL-15 iNKT cells against PSCA^+^ Capan-1, PSCA^+^ MIA Paca-2, and PSCA^+^ Aspc-1 or PSCA^–^ Panc-1 and PSCA^–^ BxPC-3 tumor cells at an E:T ratio of 1:1. Experiments were repeated with 3 donors. (**B**) Representative microscopic images show the killing as noted in **A** after 90 hours of coincubation. Experiments were repeated with 3 donors. (**C**) Freshly isolated human primary NK cells were cultured in the presence of supernatants from nontransduced iNKT (NT supernatant), sIL-15 iNKT, PSCA CAR iNKT, or PSCA CAR_sIL-15 cells for 2 days. Capan-1 cells were labeled with ^51^Cr and served as target cells. The labeled target cells were added to the cultured NK cells in the presence of respective supernatants for an additional 12 hours. The cytotoxicity levels were measured by ^51^Cr release assay. *n* = 4 donors. NT versus PSCA, *P* = 0.2218; NT versus sIL-15, *P* < 0.0001; PSCA versus PSCA CAR sIL-15, *P* < 0.0001; PSCA versus sIL-15, *P* < 0.0001; sIL-15 versus PSCA s15, *P* = 0.1157. Statistical analyses were performed by 1-way ANOVA with *P* values corrected for multiple comparisons by Bonferroni’s method.

Next, the cytolytic function of PSCA CAR_sIL-15 iNKT cells was assessed using real-time cell analysis (RTCA). PSCA CAR_sIL-15 iNKT cells demonstrated robust killing activity against PSCA^+^ tumor cell lines, including Capan-1 cells, MIA PaCa-2 cells, and Aspc-1 cells, in contrast with sIL-15 iNKT cells. However, neither sIL-15 iNKT cells nor PSCA CAR_sIL-15 iNKT cells exhibited cytotoxicity against the PSCA^–^ cell line Panc-1 and both showed relatively modest but equivalent killing against the PSCA^–^ cell line BxPC-3 (Figure 3, A and B, and Supplemental Figure 1; supplemental material available online with this article; https://doi.org/10.1172/JCI179014DS1). Collectively, our in vitro data indicate that the difference in activation, degranulation, cytokine secretion, and cytolysis between PSCA CAR_sIL-15 iNKT cells and sIL-15 iNKT cells is specific for the expression of the PSCA CAR on iNKT cells as well as for PSCA expression on the tumor cell lines.

To explore the function of sIL-15 within the construct, we measured the bystander effect of IL-15, secreted by engineered iNKT cells, on nontransduced, freshly isolated NK cells and T cells. For this purpose, the supernatants of nontransduced iNKT cells (NT), sIL-15 iNKT cells, PSCA CAR iNKT cells, and PSCA CAR sIL-15 iNKT cells were collected. Freshly isolated human NK cells or T cells were cultured in these 4 different supernatants for 2 days and the levels of NK cell and T cell cytotoxicity were measured by ^51^Cr release assay using Capan-1 cells as target cells. Compared with supernatants from the NT group, the supernatants of PSCA CAR iNKT did not activate T cells and NK cells, while the supernatants of sIL-15 iNKT cells and PSCA CAR_sIL-15 iNKT cells resulted in significantly higher levels of NK and T cell activation when compared with supernatants from the NT or PSCA (Figure 3C and Supplemental Figure 2). These data suggest that the IL-15 from these supernatants can activate NK cells and T cells via a bystander effect. Thus, IL-15 produced by our engineered NKT cells can be released into the extracellular milieu with the potential for a local effect on cell activation.

### PSCA CAR_sIL-15 iNKT cells show superior therapeutic activity in 2 in vivo PDAC metastasis models without notable toxicity.

**Figure 4:**
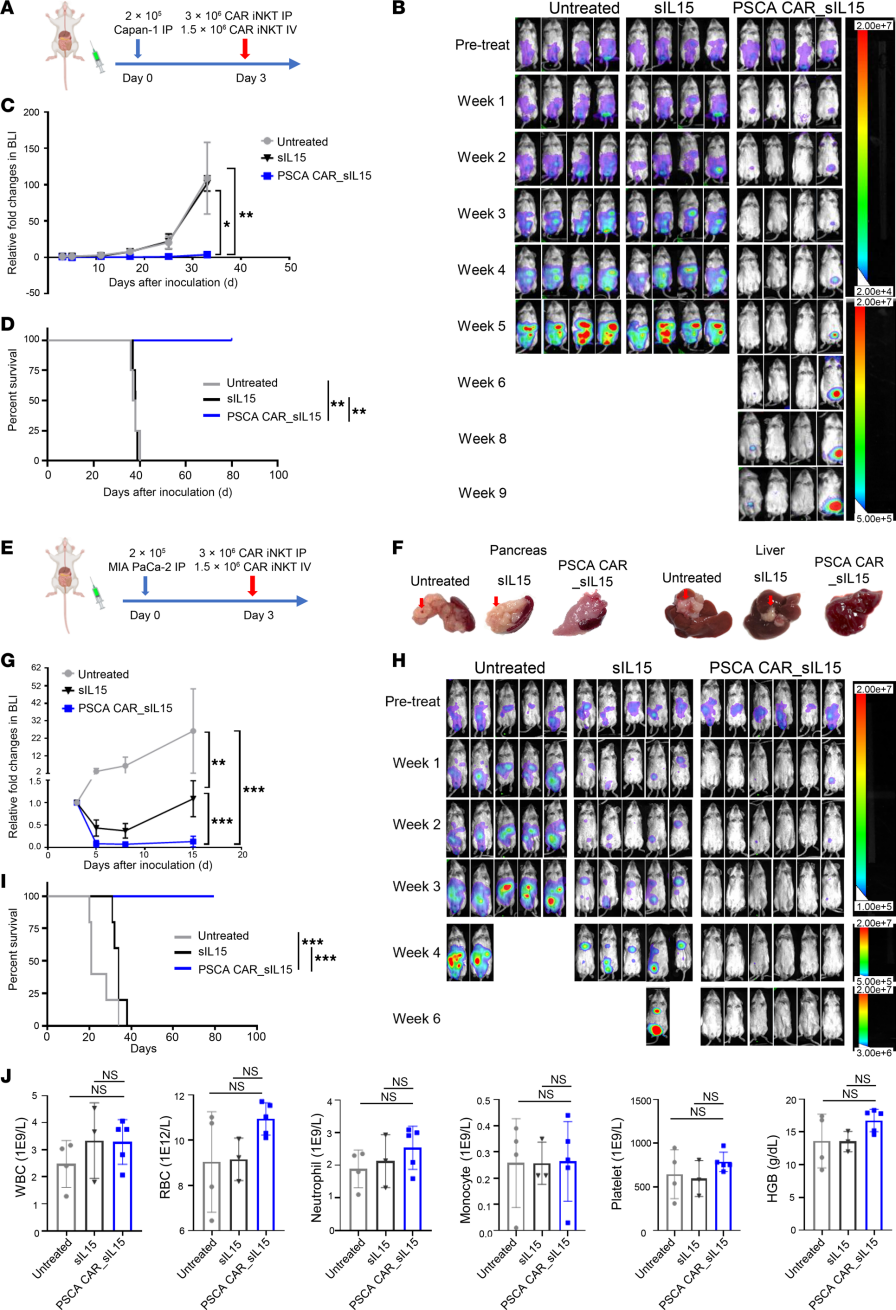
In vivo assessment of PSCA CAR_sIL-15 iNKT cells. (**A**) Treatment schema for i.p. plus i.v. injection of PSCA CAR_sIL-15 iNKT cells in a human metastatic PDAC model established by i.p. injection of PSCA^+^ Capan-1_luc cells into NSG mice. The image was created in BioRender. (**B**) Tumor growth, directly correlated with color intensity, was monitored by BLI until week 9. (**C**) Graphical depiction of BLI from **B** up to day 32. The results are displayed as mean ± SD (*n* = 4). *P < 0.05; **P < 0.01 (2-way ANOVA). (**D**) Overall Kaplan–Meier survival curve. **P < 0.01 (log-rank test, *n* = 5). Compared with the 2 control groups, PSCA CAR_sIL-15 iNKT cells significantly inhibited the progression of metastatic PDAC and prolonged the survival of the tumor-bearing mice. (**E**) Schematic diagram as in **A** but with MIA PaCa-2_luc PDAC tumor cells. (**F**) Representative images of the pancreas and liver from each treatment group at the endpoint of the in vivo experiment. Red arrows mark metastatic tumors in the liver. PSCA CAR_sIL-15 iNKT cells demonstrated strong therapeutic effects, as evidenced by their ability to kill MIA PaCa-2 cells in the pancreas and the liver. (**G**) Summary of relative fold change in BLI over 15 days as shown in **H**. The results are displayed as mean ± SD (*n* = 5). **P < 0.01; ***P < 0.001 (2-way ANOVA). (**H**) The growth of the tumor was monitored by BLI imaging until week 6. (**I**) Overall Kaplan-Meier survival curve. ***P < 0.001 (log-rank test, *n* = 5). PSCA CAR_sIL-15 iNKT cells completely eradicated PDAC in vivo. sIL-15 iNKT cells were inferior to PSCA CAR_sIL-15 iNKT cells but also exhibited some degree of efficacy in delaying tumor progression in mice bearing MIA PaCa-2 cells. (**J**) Assessment of blood cells and HGB on day 15 after PDAC cell transplantation (12 days after treatment with PBS, sIL-15 iNKT cells, or PSCA CAR_sIL-15 iNKT cells). Values represent mean ± SD (*n* = 5). Not significant (2-way ANOVA).

To validate the in vitro antitumor effectiveness of PSCA CAR_sIL-15 iNKT cells noted above, we established 2 PDAC metastatic orthotopic models for in vivo study. Previously, we demonstrated that a combination of i.p. and i.v. injections of PSCA CAR NK cells killed PSCA^+^ Capan-1 cells in the pancreas and those that metastasized to the liver and lung (22). Therefore, we combined i.p. and i.v. injections of PSCA CAR_sIL-15 iNKT cells to treat PDAC tumor-bearing mice. The procedure for establishing the Capan-1 cell–based metastatic PDAC model and the treatment are depicted in Figure 4A. Briefly, 2 × 10^5^ PSCA^+^ Capan-1 cells expressing the firefly luciferase (FFL) gene were i.p. injected into NOD-scid IL2Rgamma^null^ (NSG) mice on day 0. Three days after tumor implantation, mice were treated with a single dose of 3 × 10^6^ PSCA CAR_sIL-15 iNKT cells by i.p. injection combined with 1.5 × 10^6^ PSCA CAR_sIL-15 iNKT cells by i.v. injection. Saline and sIL-15 iNKT cells were administered through the same routes as control. Progression of tumors was monitored by bioluminescence (BLI) until week 9, and survival data were recorded. Compared with the 2 control groups, PSCA CAR_sIL-15 iNKT cells significantly inhibited the progression of metastatic PDAC and significantly prolonged the survival of the tumor-bearing mice, reaching 100% survival by day 80, while allowing for maintenance of body weight compared with control mice in vivo (Figure 4, B–D, and Supplemental Figure 3). We also compared the antitumor effects in vivo of i.p. plus i.v., i.p. alone, and i.v. alone. For this purpose, mice were injected with 5 × 10^5^ Capan-1-luc cells on day 1. On day 7, mice were randomly divided into 4 groups: group 1, PBS; group 2, i.p. injection of 4 × 10^6^ PSCA CAR_sIL-15 iNKT cells per mouse; group 3, i.v. injection of 4 × 10^6^ PSCA CAR_sIL-15 iNKT cells per mouse; group 4, i.p. injection of 2 × 10^6^ plus i.v. injection of 2 × 10^6^ of PSCA CAR_sIL-15 iNKT cells per mouse. Tumor burden and survival were monitored as above. Group 2 (i.p. injection) showed significantly greater survival compared with group 3 (i.v. injection). Furthermore, there was no difference in survival between group 2 and group 4 (the median survival day of group 2 was 71 days versus the median survival of 77.5 days for group 4) (Supplemental Figure 4).

We constructed another metastatic PDAC model using the PSCA^+^ cell line MIA PaCa-2 cells to confirm the therapeutic capability of PSCA CAR_sIL-15 iNKT cells depicted in Figure 4E. In this model, PSCA CAR_sIL-15 iNKT cells also demonstrated a strong therapeutic effect, as evidenced by their ability to kill MIA PaCa-2 cells in the pancreas and the liver (Figure 4F), completely eradicate PDAC in vivo, maintain remission, and significantly extend survival compared with the untreated and sIL-15 iNKT–treated groups (Figure 4, G–I). Furthermore, a hematological analysis of blood samples from treated mice revealed that PSCA CAR_sIL-15 iNKT cell treatment had no significant adverse effect on blood cell counts and hemoglobin (HGB) levels when compared with both the untreated group and the sIL-15 iNKT cell group (Figure 4J).

### PSCA CAR_sIL-15 iNKT cells exert superior therapeutic activity in an orthotopic PDAC model without notable toxicity.

**Figure 5:**
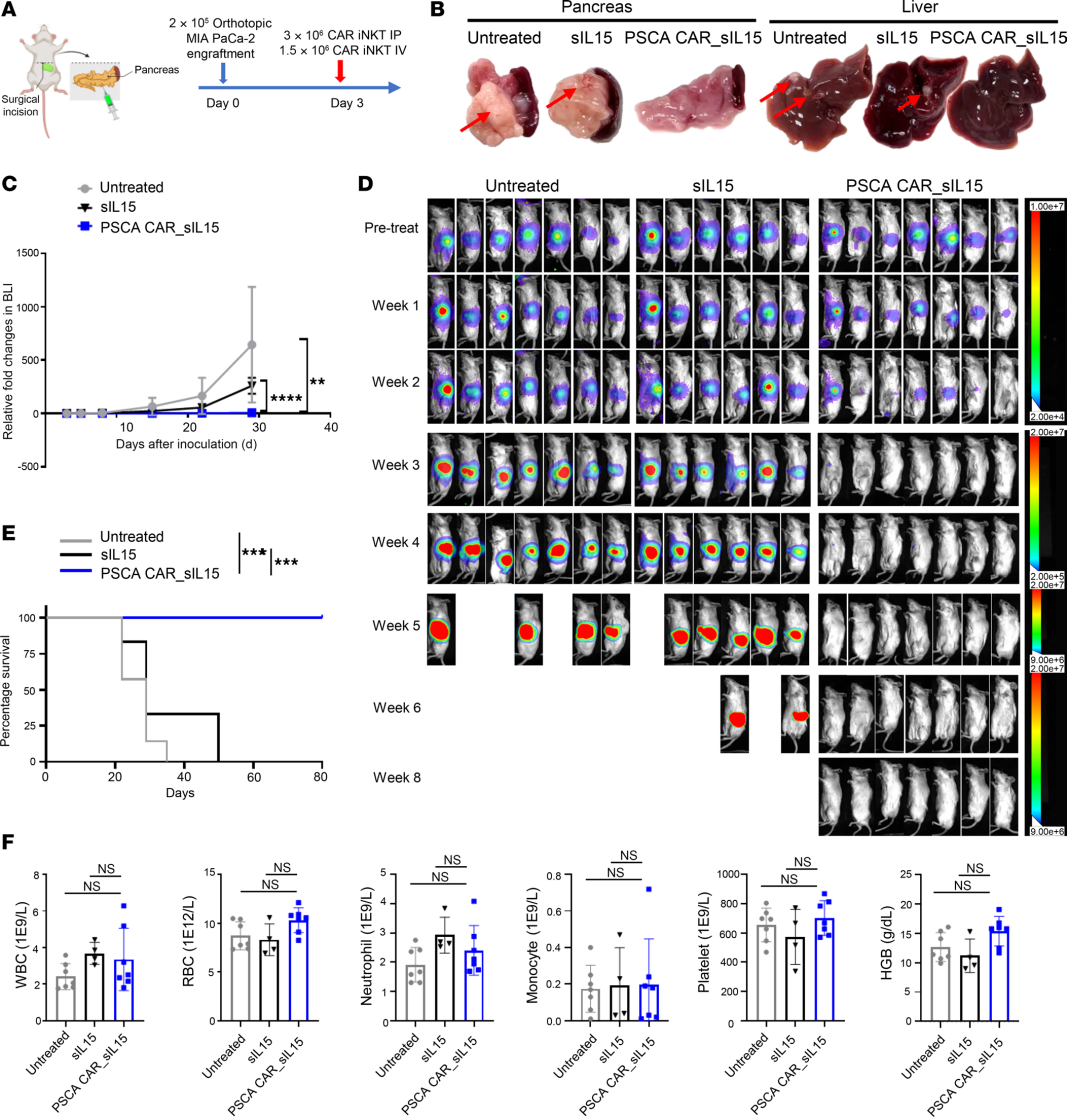
PSCA CAR_sIL-15 iNKT cells eliminate human PDAC cells in an orthotopic tumor model and maintain long-term tumor-free survival. (**A**) Schematic diagram of treatment with PSCA CAR_sIL-15 iNKT cells in a human orthotopic PDAC model established by i.p. injection of MIA PaCa-2_luc cells into NSG mice. The image was created in BioRender. (**B**) Representative images of the pancreas and the liver of each group in the MIA PaCa-2-transplanting PDAC mouse model at the endpoint of the in vivo experiments. Red arrows mark metastatic tumors in the liver. In this orthotopic PDAC model, PSCA CAR_sIL-15 iNKT cells efficiently eliminated carcinoma in situ within the pancreas and decreased metastatic lesion formation in the liver. (**C**) Summary statistical data of mouse tumor burden changes of each treatment group. The results are displayed as mean ± SD. **P < 0.01; ****P < 0.0001 (2-way ANOVA). *n* = 7 for the untreated and PSCA CAR_sIL-15 groups. *n* = 6 for the sIL-15 group. (**D**) The growth of the tumor was monitored by BLI imaging until week 8. (**E**) Overall Kaplan-Meier survival curve. ***P < 0.001 (log-rank test). *n* = 7 for the untreated and PSCA CAR_sIL-15 groups. *n* = 6 for the sIL-15 group. Treatment with PSCA CAR_sIL-15 iNKT cells resulted in complete clearance of orthotopic tumors and reached 100% survival. (**F**) Assessment of blood cell populations on day 15 after PDAC cell transplantation (12 days after iNKT cell treatment). Peripheral blood counts and HGB in the PSCA CAR_sIL-15 iNKT group were not changed compared with the untreated group and sIL-15 iNKT cell treatment group. Values represent mean ± SD (*n* = 7 for the untreated and PSCA CAR_sIL-15 groups. *n* = 4 for the sIL-15 group.). 2-way ANOVA.

We then conducted an extensive evaluation of PSCA CAR_sIL-15 iNKT cells in an orthotopic PDAC model (Figure 5A). This model showed locoregional cancer cell spread and liver metastasis, thus mimicking the condition of PDAC patients. To establish the model, 2 × 10^5^ FFL-expressing MIA PaCa-2 cells were injected intrapancreatically on day 0. On day 3, mice received a single dose of 3 × 10^6^ PSCA CAR_sIL-15 iNKT cells by i.p. injection and 1.5 × 10^6^ PSCA CAR_sIL-15 iNKT cells by i.v. injection. In this orthotopic PDAC model, PSCA CAR_sIL-15 iNKT cells efficiently eliminated macroscopic evidence of MIA PaCa-2 cells in situ within the pancreas and decreased metastatic lesion formation in the liver (Figure 5B). Treatment with PSCA CAR_sIL-15 iNKT cells resulted in complete macroscopic clearance of orthotopic tumors and reached 100% survival at 80 days (Figure 5, C–E) without affecting peripheral blood counts and HGB compared with the untreated group and sIL-15 iNKT cell treatment group (Figure 5F).

### Gemcitabine-resistant PDAC can be overcome by PSCA CAR_sIL-15 iNKT cells.

**Figure 6:**
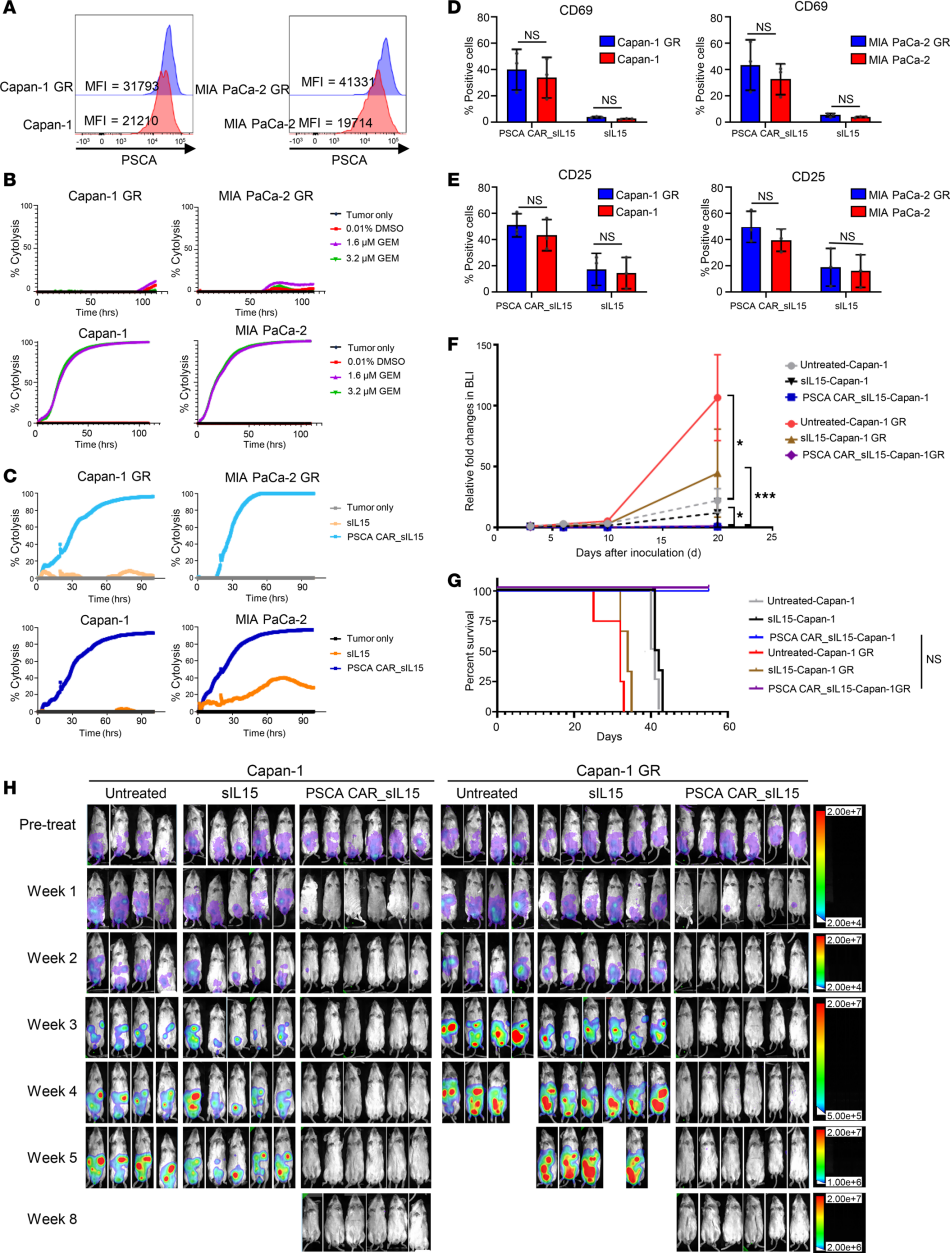
PSCA CAR_sIL-15 iNKT cells overcome gemcitabine resistance in PDAC. (**A**) MFI of PSCA (blue solid histograms) on GR cell lines (Capan-1 GR and MIA Paca-2 GR) compared with parental cell lines (red solid histograms) as measured flow cytometry. (**B**) Cytotoxicity of gemcitabine measured by RTCA in the presence of different concentrations of gemcitabine on GR and parental PDAC cell lines. Capan-1 GR and MIA Paca-2 GR were not while their parental cell lines were killed by gemcitabine at the concentrations of 1.6 μM and 3.2 μM. (**C**) Cytotoxicity of sIL-15 iNKT cells and PSCA CAR_sIL-15 iNKT cells against GR PDAC cell lines (Capan-1 GR and MIA Paca-2 GR) and the parental PDAC cell lines at an E:T ratio of 1:2, measured by RTCA assay. PSCA CAR_sIL-15 iNKT cells maintained their potent killing ability against Capan-1 GR and MIA PaCa-2 GR cells compared with the parental cells. (**A**–**C**) Experiments were repeated 3 times or with 3 different donors. Expression of T cell activation markers CD69 (**D**) and CD25 (**E**) on sIL-15 iNKT cells and PSCA CAR_sIL-15 iNKT cells following a 24-hour coincubation with Capan-1 GR, Capan-1, MIA PaCa-2 GR, or MIA PaCa-2 (gated on EGFR^+^ cells). Data are represented as mean ± SD (*n* = 3). There were comparable levels of CD69 and CD25 expression in PSCA CAR_sIL-15 iNKT cells when they were coincubated with the GR cells and the parental cells. (**F**) The parent cell lines Capan-1_luc and Capan-1 GR_luc were injected i.p. (2 × 10^5^ cells/mouse). Three days later, tumor engraftment was confirmed by BLI and visually displayed in vivo weekly up to 8 weeks. The fold changes of BLI for each treatment group were measured. *n* = 4 for the untreated group. *n* = 5 for the sIL-15 group. *n* = 6 for the PSCA CAR_sIL-15 group. (**G**) Overall Kaplan-Meier survival curve. log-rank test (*n* = 4 for the untreated group. *n* = 5 for the sIL-15 group. *n* = 6 for the PSCA CAR_sIL-15 group.). (**H**) The growth of the tumor was monitored by BLI imaging until week 8. A single dose of PSCA CAR_sIL-15 iNKT cells still significantly suppressed Capan-1 GR tumor progression, and the antitumor effect of CAR_sIL-15 iNKT cells was not different when compared with the Capan-1 mouse tumor.

Gemcitabine-based therapy is a standard first-line therapy for patients with advanced PDAC (24). However, invariable tumor recurrence after gemcitabine results in relapse and progression, allowing for reduced patient survival (25). Therefore, we next determined whether PSCA CAR_sIL-15 iNKT cells can kill gemcitabine-resistant (GR) PDAC in vitro and in vivo. In this scenario, we generated 2 GR cell lines (Capan-1 GR and MIA Paca-2 GR) by exposing parental Capan-1 and MIA PaCa-2 cells to escalating concentrations of gemcitabine for 9 months, as previously reported (26). The GR cell lines, Capan-1 GR and MIA PaCa-2 GR, showed a modest increase in the expression of PSCA compared with parental Capan-1 and MIA PaCa-2 cells (Figure 6A). Besides, Capan-1 GR and MIA PaCa-2 GR were not killed by gemcitabine at concentrations of 1.6 μM and 3.2 μM, while the same concentration of gemcitabine eradicated all parental or unresistant Capan-1 and PaCa-2 cells (Figure 6B, Supplemental Figure 5, and Supplemental Figure 6). We first explored the cytolytic effect of PSCA CAR_sIL-15 iNKT cells targeting Capan-1 GR and MIA PaCa-2 GR cell lines in vitro. RTCA results indicated that PSCA CAR_sIL-15 iNKT cells had similar potent killing of Capan-1 GR and MIA PaCa-2 GR cell lines compared with their killing of the parental cell lines (Figure 6C). Additionally, we demonstrated equivalent expression levels of CD25, CD69, CD107a, TNF-α, and IFN-γ in PSCA CAR_sIL-15 iNKT cells after coincubation with GR cell lines or their parental counterparts (Figure 6, D and E, and Supplemental Figure 7). Next, we evaluated the therapeutic effect of PSCA CAR_sIL-15 iNKT cells against the Capan-1 GR cell line in vivo. The parental Capan-1 cell line was injected as a control. Compared with the parental Capan-1 cell line, the Capan-1 GR cell line was found to have a slightly more rapid progression with shorter survival time in vivo in the absence of gemcitabine (Figure 6, F–H). In in vivo mouse tumor models, a single dose of PSCA CAR_sIL-15 iNKT cells provided comparable suppression of the Capan-1 GR cell line growth when compared with the parental cell line (Figure 6, F–H). Taken together, PSCA CAR_sIL-15 iNKT cells exerted equivalent in vitro and in vivo killing of PSCA^+^ tumors regardless of whether it was against GR PDAC cells or their parental cell lines, suggesting that PSCA CAR_sIL-15 iNKT cells may have the potential to be used against GR PDAC in patients.

### Cryopreserved PSCA CAR_sIL-15 iNKT cells exhibit antitumor function comparable to fresh PSCA CAR_sIL-15 iNKT cells.

**Figure 7:**
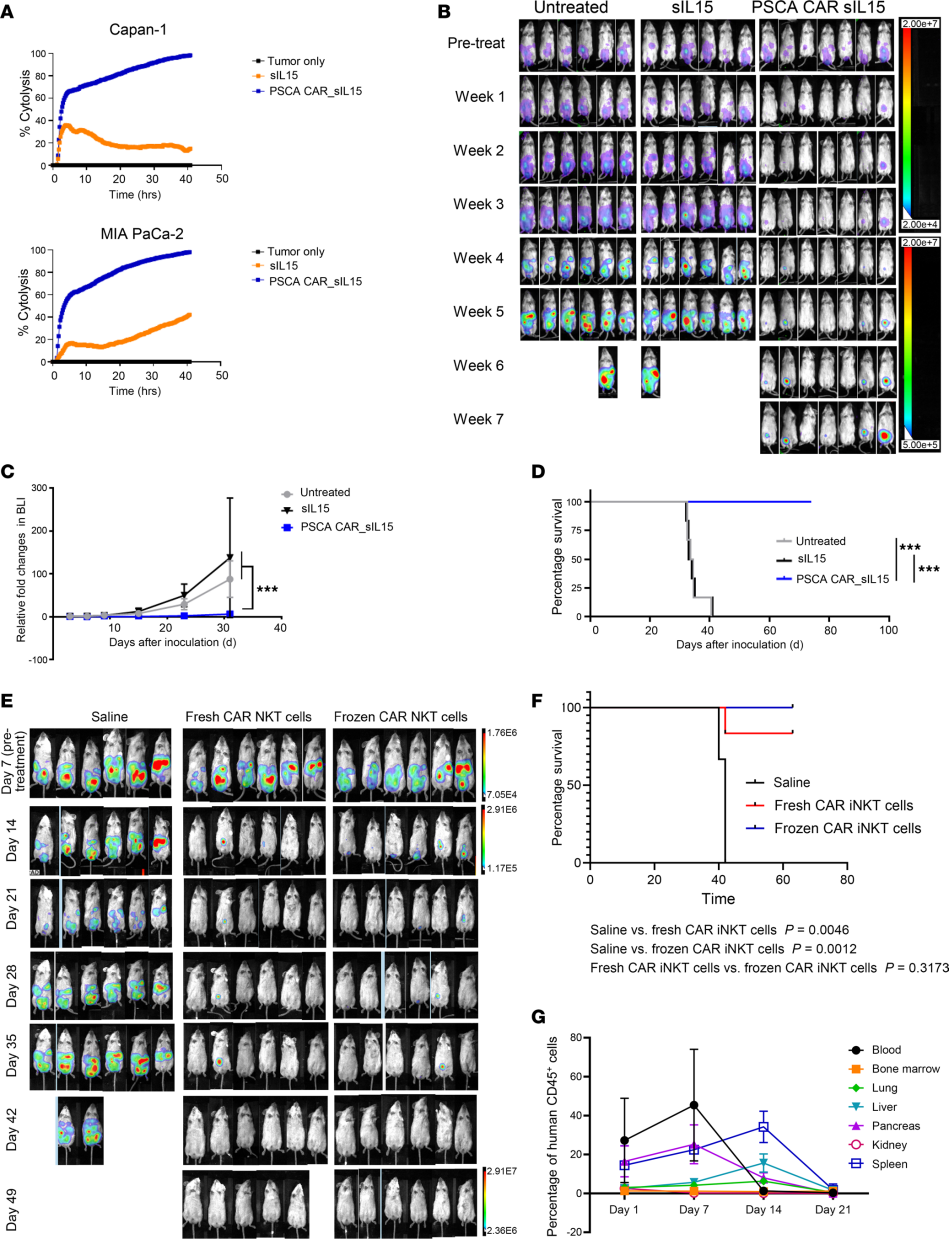
Cryopreserved and fresh PSCA CAR_sIL-15 iNKT cells exhibit comparable antitumor activity. (**A**) In vitro cytotoxicity of off-the-shelf sIL-15 iNKT cells and off-the-shelf PSCA CAR_sIL-15 iNKT cells against PSCA^+^ PDAC cell lines, measured by RTCA at an E:T ratio 1:1. Experiments were repeated with 3 donors. (**B**) In vivo assessment of Capan-1 tumor burden was monitored by BMI during treatment with off-the-shelf cryopreserved sIL-15 iNKT cells and off-the-shelf cryopreserved PSCA CAR_sIL-15 iNKT cells until week 7. (**C**) Graphical summary of mouse tumor burden during treatment shown in **B**. Data are displayed as mean ± SD until day 30. ***P < 0.001 (2-way ANOVA). *n* = 6 for the untreated and sIL-15 groups. *n* = 7 for the PSCA CAR_sIL-15 group. (**D**) Overall Kaplan-Meier survival curve as the result of treatment shown in **B**. ***P < 0.001 (log-rank test). *n* = 6 for the untreated and the sIL-15 groups. *n* = 7 for the PSCA CAR_sIL-15 group. (**E**) NSG mice were injected with 5 × 10^5^ Capan-1-luc cells on day 1. On day 7, mice received fresh PSCA CAR_sIL-15 iNKT cells or frozen PSCA CAR_sIL-15 iNKT cells (i.p. 4 × 10^6^ and i.v. 2 × 10^6^ /mouse, respectively). Tumor burden was monitored by BLI until day 49. (**F**) Overall Kaplan-Meier survival curve resulting from **E**. *n* = 6 per group. One mouse receiving fresh PSCA CAR_sIL-15 iNKT cells died accidentally during imaging. (**G**) NSG mice were injected with 5 × 10^5^ Capan-1-luc cells on day 1. On day 7, mice were injected with off-the-shelf cryopreserved PSCA CAR-sIL-15 iNKT cells (i.p. 4 × 10^6^ plus i.v. 2 × 10^6^/mouse). Blood, bone marrow, lung, liver, pancreas, kidney, and spleen were harvested on days 1, 7, 14, and 21 after PSCA CAR_sIL-15 iNKT cell injection. PSCA CAR_sIL-15 iNKT cell persistence was detected by flow cytometry using hCD45^+^ (*n* = 3 or 4 mice per time point).

Off-the-shelf allogeneic CAR-based products substantially shorten the production time, increase the distribution, and reduce the cost of CAR-based cell therapies. We validated the in vitro and in vivo antitumor potential of readily available, cryopreserved, and off-the-shelf PSCA CAR_sIL-15 iNKT cells generated in our laboratory. PSCA CAR_sIL-15 iNKT cells were recovered from cryopreservation and still displayed robust cytotoxicity against Capan-1 and MIA PaCa-2 cells measured by RTCA in vitro (Figure 7A). Subsequently, we examined the in vivo antitumor activity of readily available frozen-thawed off-the-shelf PSCA CAR_sIL-15 iNKT cells in the Capan-1 cell established metastatic PDAC model. The results showed that these off-the-shelf cryopreserved PSCA CAR_sIL-15 iNKT cells maintained their therapeutic effectiveness in impeding the progression of metastatic PDAC and prolonged the survival time of mice (Figure 7, B–D).

We assessed the antitumor in vitro and in vivo activity of freshly produced PSCA CAR_sIL-15 iNKT cells and off-the-shelf cryopreserved PSCA CAR_sIL-15 iNKT cells generated from the same donor in a head-to-head comparison. First, the cytotoxicity of freshly produced PSCA CAR iNKT cells and off-the-shelf cryopreserved PSCA CAR iNKT cells was measured against Capan-1 cells. This was found to be equivalent at high effector/target ratios (16:1 and 8:1). The cytotoxicity of the off-the-shelf cryopreserved PSCA CAR iNKT cells was found to be moderate but significantly decreased at low ratios (4:1 and 2:1) compared with that of fresh PSCA CAR iNKT cells when targeting Capan-1 cells. (Supplemental Figure 8A).

We compared the in vivo antitumor ability of the fresh CAR iNKT cells and off-the-shelf cryopreserved CAR iNKT cells in a metastatic PDAC model established by Capan-1 cells. Fresh CAR iNKT cells and off-the-shelf cryopreserved CAR iNKT cells showed equivalent antitumor activity and both showed significantly stronger antitumor activity compared with the saline group (Figure 7, E and F).

Next, we measured the persistence and biodistribution of off-the-shelf cryopreserved CAR iNKT cells in the metastatic PDAC model. On days 1, 7, 14, and 21 after PSCA CAR_sIL-15 iNKT cell treatment, the mice were euthanized. Blood, bone marrow, lung, liver, pancreas, kidney, and spleen were harvested and the percentages of PSCA CAR_sIL-15 iNKT cells among mononuclear cells (hCD45^+^ cells) were analyzed by flow cytometry. At the early time points (days 1 and 7 after PSCA CAR_sIL-15 iNKT cell injection), PSCA CAR_sIL-15 iNKT cells were mainly in the blood, pancreas, and spleen. Fourteen days after PSCA CAR_sIL-15 iNKT cell injection, PSCA CAR_sIL-15 iNKT cells existed in the spleen, liver, pancreas, and lung. Twenty-one days after PSCA CAR_sIL-15 iNKT cell injection, PSCA CAR_sIL-15 iNKT cells could be still detectable in the liver and spleen but at very low percentages (Figure 7G).

We also detected the proliferation of PSCA CAR_sIL-15 iNKT cells in vivo. For this purpose, expanded PSCA CAR_sIL-15 iNKT cells from the cryopreserved product were labeled with CFSE before injection. The mice engrafted with Capan-1 cells were treated with CFSE-labeled PSCA CAR_sIL-15 iNKT cells. Lung, liver, pancreas, and spleen were harvested 2 days after the PSCA CAR_sIL-15 iNKT cell injection. PSCA CAR_sIL-15 iNKT cell proliferation was detected by flow cytometry using hCD45^+^ CFSE. Compared with the high CFSE fluorescence intensity or low proliferative rate of preinjected PSCA CAR_sIL-15 iNKT cells, the CFSE fluorescence intensity was significantly decreased in the CAR_sIL-15 iNKT cells from lung, liver, pancreas, and spleen, indicating that PSCA CAR_sIL-15 iNKT cells are proliferating in vivo (Supplemental Figure 8B).

Finally, we measured the biodistribution of the IL-15 from PSCA CAR_sIL-15 iNKT cells in the metastatic PDAC model by quantitative reverse-transcriptase PCR (RT-qPCR). Four days after PSCA CAR_sIL-15 iNKT cell injection, we collected the liver, pancreas, and spleen. The mRNA expression levels of IL-15 were significantly higher in the pancreas, liver, and spleen when compared with the PBS-injected group (Supplemental Figure 8C).

### Cryopreserved PSCA CAR_sIL-15 iNKT cells exhibit antitumor function comparable to conventional PSCA CAR_sIL-15 T cells, but the former do not cause GvHD or cytokine release syndrome.

**Figure 8:**
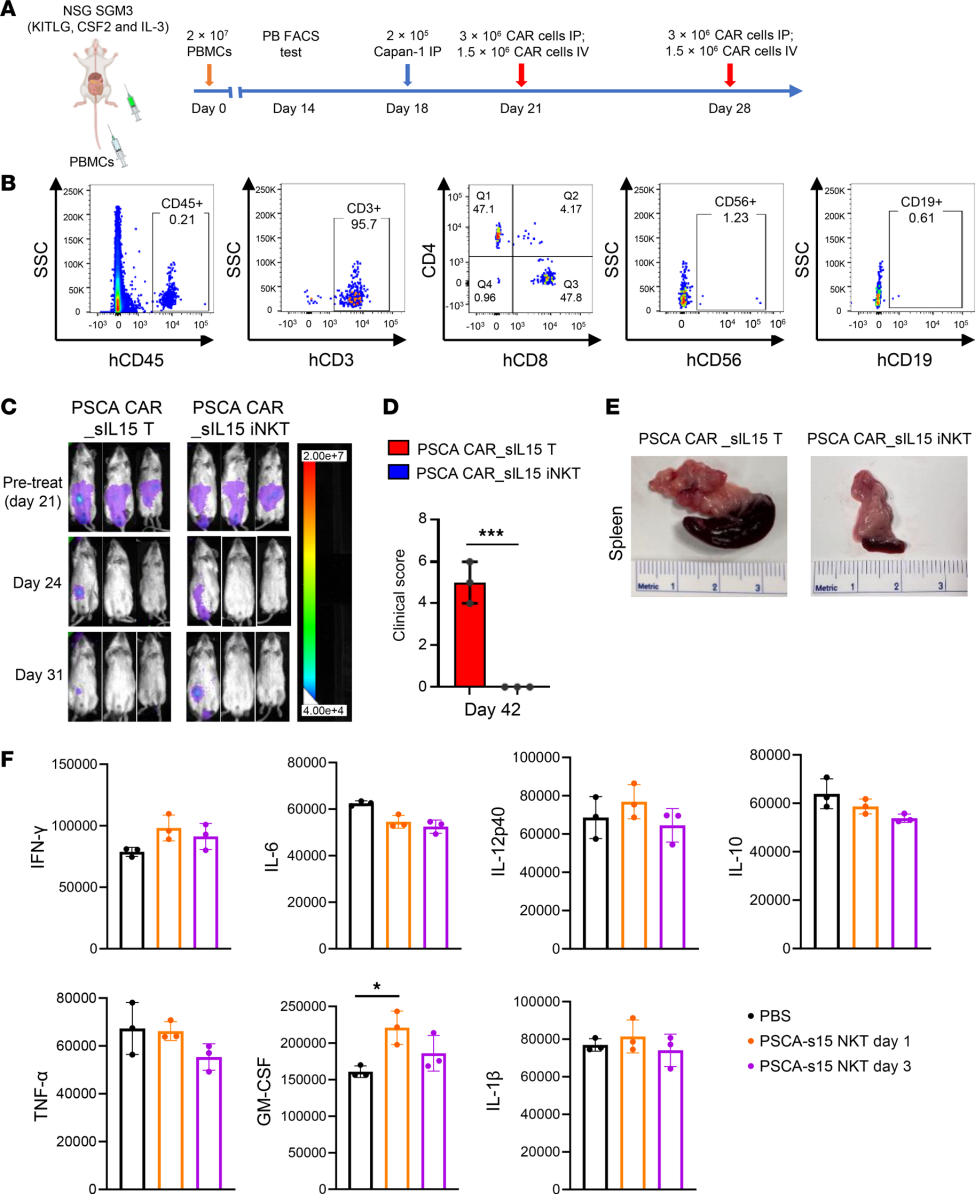
Cryopreserved PSCA CAR_sIL-15 iNKT cells exhibit anti-tumor functions comparable to conventional PSCA CAR T cells but do not cause GvHD. (**A**) Schematic diagram of treatment with PSCA CAR_sIL-15 iNKT cells or PSCA CAR_sIL-15 T cells in a Capan-1–transplanted metastatic PDAC humanized mouse model to investigate the risk of GvHD. (**B**) Fourteen days after transplantation, the percentage of repopulated human CD3^+^CD4^+^ T cells, CD3^+^CD8^+^ T cells, CD19^+^ B cells, and CD56^+^ NK cells were assessed in the peripheral blood of the mice. (**C**) The burden of tumor was monitored by BLI on days 24 and day 31. (**D**) Clinical GvHD scores were observed on day 42. ***P < 0.001. *n* = 3 per group. (**E**) Splenic dimensions of tumor-bearing mice treated with PSCA CAR_sIL-15 T cells (left) or PSCA CAR_sIL-15 iNKT cells (right) measured on day 42. (**F**) SGM3 mice engrafted with PBMCs were injected with 2 × 10^6^ Capan-1-luc cells. Seven days later, mice were injected either PBS (*n* = 3) or off-the-shelf cryopreserved iNKT cells (4 × 10^6^ cells/mouse i.p. plus 2 × 10^6^ cells/mouse i.v., *n* = 6). Sera were harvested 1 (day 1) and 3 days (day 3) after the iNKT cell injection to assess for CRS-associated cytokines. The sera from the PBS group were collected 1 day after injection (Day 1). Data are displayed as the mean ± SD. Statistical analyses were conducted using a 2-sided *t* test.

We tested the safety of readily available, off-the-shelf PSCA CAR_sIL-15 iNKT cells for the risk of developing GvHD and the cytokine release syndrome (CRS). To investigate the risk of GvHD associated with off-the-shelf PSCA CAR_sIL-15 iNKT cell therapy compared with PSCA CAR_sIL-15 T cell therapy, NSG SGM3 mice received 2 × 10^7^ human PBMCs via the tail vein to establish humanized mice on day 0 (Figure 8A). Fourteen days after transplantation, repopulated human CD3^+^CD4^+^ T cells, CD3^+^CD8^+^ T cells, CD19^+^ B cells, and CD56^+^ NK cells were detected in blood, consistent with successful engraftment (Figure 8B). The humanized mice were inoculated i.p. with 2 × 10^5^ Capan-1 cells on day 18. On days 21 and 28, they were administered with either allogeneic, cryopreserved off-the-shelf PSCA CAR_sIL-15 iNKT cells or cryopreserved off-the-shelf PSCA CAR_sIL-15 T cells (3 × 10^6^ CAR^+^ cells i.p., 1.5 × 10^6^ CAR^+^ cells i.v.; derived from the same donor) were administered twice on days 21 and 28 (Figure 8A). Tumor progression was monitored by BLI until day 31, and the results showed that there was no significant difference in the antitumor efficacy between PSCA CAR_sIL-15 iNKT cells and PSCA CAR_sIL-15 T cells, as both cells significantly suppressed tumor progression in mice (Figure 8C). Clinical GvHD scores were monitored in a blinded fashion by evaluating systemic symptoms (27), including weight loss, posture, activity, fur texture, and skin integrity. On day 42, PSCA CAR_sIL-15 T cell treatment showed significantly higher GvHD scores when compared with PSCA CAR_sIL-15 iNKT cells (Figure 8D). Tumor-bearing mice treated with PSCA CAR_sIL-15 T cells developed splenomegaly, while PSCA CAR_sIL-15 iNKT cell–treated mice had normal spleen size (Figure 8E). We next measured CRS-related cytokines in the humanized NSG-SGM3 mice bearing metastatic PDAC treated with PSCA CAR_sIL-15 iNKT cells. One day and 3 days after treatment, mice were euthanized, and sera were harvested for multiplex analysis for CRS-related cytokines (28). PBS was injected as a control. PSCA CAR_sIL-15 iNKT cells did not induce the release of CRS-related cytokines IFN-γ, IL-6, IL-12, IL-10, TNF-α, or IL-1β, while GM-CSF modestly and transiently increased compared with PBS (Figure 8F and Supplemental Figure 9).

Hence, unlike PSCA CAR_sIL-15 T cells, which induced lethal GvHD and CRS in humanized NSG-SGM3 mice, iNKT cells expressing the same CAR construct exhibited comparable antitumor effectiveness without causing substantial GvHD or CRS.

## Discussion

In this study, we show that PSCA CAR_sIL-15 iNKT exerted excellent in vitro expansion and potent antitumor activity against human PDAC^+^ cell lines, with very little if any phenotypic evidence of exhaustion. We demonstrate that PSCA CAR_sIL-15 iNKT cells exert strong therapeutic activity in both metastatic and orthotopic PDAC models in vivo without causing notable toxicity. In addition, PSCA CAR_sIL-15 iNKT cells have the potential to overcome GR PDAC in vitro and in vivo. Off-the-shelf cryopreserved PSCA CAR_sIL-15 iNKT cells demonstrate an efficacy comparable to freshly made PSCA CAR_sIL-15 iNKT cells in the same PDAC model. Most importantly, readily available off-the-shelf cryopreserved PSCA CAR_sIL-15 iNKT cells maintained excellent antitumor activities without causing CRS and GvHD.

Although it has been suggested that iNKT cells traffic to solid tumors in response to chemokines produced by tumor cells, stromal cells, and TAM (12) and iNKT cell infiltration into primary tumors correlates with better outcomes in different tumors (13), our results suggest NKT cells without constructing PSCA CAR show very limited efficacy against PDAC in multiple PDAC models. The absence of efficacy in naked iNKT cells or sIL-15 iNKT cells could be explained by failure to traffic into a highly immunosuppressive TME characterized by dense desmoplasia and abundant infiltration of immunosuppressive cells. In the current study, we show that the engineered PSCA CAR_sIL-15 iNKT cells can overcome these barriers and show strong cytotoxicity against PDAC^+^ tumor cells both in vitro and in vivo.

CD1d-restricted iNKT cells can directly kill CD1d^+^ tumor cells through CD1d recognition. It is possible that PSCA CAR_sIL-15 iNKT cells may achieve a similar effect against CD1d^+^ immune suppressor cells. Notably, PDAC patients exhibited heightened infiltration of myeloid-derived suppressor cells (MDSCs), which express CD1d (29), within pancreatic tumors (30). Elevated levels of MDSCs in PBMCs from PDAC patients independently predict shorter survival (31). Similarly, heightened numbers of TAM independently correlated with reduced overall survival in PDAC patients (32, 33). On the other hand, CD1d-restricted iNKT cells play a pivotal role in tumor immunosurveillance (34, 35), and mounting evidence supports their crucial involvement in impeding tumor progression by inducing proinflammatory and immunostimulatory programs in myelomonocytic cells, irrespective of CD1d expression by cancer cells (36, 37). Moreover, in solid tumors and hematological malignancies, human and mouse CD1d-restricted iNKT cells reshape the TME by selectively eliminating CD1d-expressing protumor M2-like macrophages while preserving antitumor M1-like populations (35, 38, 39). In addition, CD1d-restricted iNKT cells can promote the maturation of MDSCs into functional antigen-presenting cells that stimulate antitumor T cell and NK cell responses (34, 40, 41). Notably, tumor-specific T cell receptor–engineered (TCR-engineered) CD1d-restricted iNKT cells induced robust antitumor responses by simultaneously targeting cancer cells and MDSCs in B16-Ova or MC38-Ova tumor-bearing mice (42). Our study demonstrates that adding sIL-15 to these powerful immunoregulatory and cytotoxic effector cellumor progression. We show this is achieved not only by direct tumor cell lysis but also by changing the “cold” TME to a “hot” one with the secretion of sIL-15. This is further supported by our data showing that coculturing PSCA CAR_sIL-15 iNKT cells with either gemcitabine-sensitive or GR PSCA^+^ tumor cells boosts secretion of granzyme B, IFN-γ, and TNF-α when compared with control sIL-15 iNKT cells.

PDAC is frequently diagnosed at an advanced stage with local and/or distant metastases leading to the poor prognosis of this disease. Gemcitabine (2′,2′-difluorodeoxycytidine) is a nucleoside analog that represents a first-line therapy to treat advanced PDAC, but relapse is inevitable, overall survival remains poor, and there are limited options for patients who have failed gemcitabine-based therapy (3, 4). The strategies for overcoming gemcitabine resistance have been vigorously investigated in the field. Our preclinical study suggests a viable alternative to overcome gemcitabine resistance, possibly by a direct non–cross-resistant mechanism of cytotoxicity as well as by modulating the immunosuppressive TME. Hypothetically, combined infusion of allogeneic, off-the-shelf PSCA CAR_sIL-15 iNKT cells with gemcitabine as the first-line therapy for advanced PDAC might prove more effective than using gemcitabine alone in improving tumor responses, disease-free survival, and overall survival. It is conceivable that sufficient numbers of PSCA CAR_sIL-15 iNKT cells trafficking to sites of primary and metastatic disease could promote MDSC maturation and polarization into functional antigen-presenting cells that stimulate antitumor T cell and NK cell responses, especially in the presence of locally secreted sIL-15. Furthermore, they may eliminate CD1d-expressing protumor M2-like macrophages while preserving antitumor M1-like populations, ultimately promoting an antitumorigenic TME. We would also propose that PSCA CAR_sIL-15 iNKT cells be considered as part of the first-line therapy because of their high specificity and excellent efficacy in targeting PSCA^+^ PDAC cells with seemingly low toxicity. We believe this approach merits clinical evaluation. The majority of PDAC patients are reported to express a low tumor mutational burden and with the exception of those PDAC patients with a high tumor mutational burden, immune checkpoint inhibitory (ICI) therapy has thus far proven ineffective in this disease (43). However, there is preclinical evidence that IL-15 can enhance ICI in solid tumors (44), and thus it might also be important to investigate PSCA CAR_IL-15 iNKT cells in combination with ICI therapy.

Our current study supports the concept that CAR iNKT cells combine the efficacy of conventional CAR T cells and the safety of CAR NK cells. We showed that PSCA CAR_sIL-15 iNKT cells have antitumor activity comparable to PSCA CAR T cells when each of these 2 PSCA CAR effector cells was administered at the same dose. However, administration of PSCA CAR_sIL-15 T cells resulted in GvHD in our tumor model, while PSCA CAR_sIL-15 iNKT cells did not. These data are consistent with earlier studies showing that donor-derived iNKT cells can effectively inhibit GvHD while preserving their antitumor activity (14, 15). In our own prior study, repeated administration of PSCA CAR_sIL-15 NK cells showed substantial anti-PDAC efficacy (22). However, we administered off-the-shelf cryopreserved PSCA CAR_sIL-15 NK cells multiple times, yet all mice eventually died of recurrent PDAC. In contrast, in the current study, we demonstrated that a single infusion of off-the-shelf cryopreserved PSCA CAR_sIL-15 iNKT cells can achieve 100% survival in similar PDAC tumor models. It is noteworthy that neither the treatment with PSCA CAR_sIL-15 NK cells nor PSCA CAR_sIL-15 iNKT cells induced notable toxicity or GvHD. Importantly, our research demonstrates that both CAR NK cells and CAR iNKT cells can be manufactured using an optimized expansion platform and stored as an off-the-shelf cryopreserved product. Essentially, our studies support the development of both cells, either separately or in combination, for clinical evaluation in treating PSCA^+^ PDAC. Since both NK cells and iNKT cells are safe, can be activated, expanded, frozen, and thawed while maintaining strong antitumor activity, and can be used in an allogeneic manner, there may be no need to separate out these 2 cell types during a manufacturing process to develop a mixture cell product for treating PDAC patients.

In conclusion, our current study demonstrates that allogeneic, off-the-shelf cryopreserved PSCA CAR_sIL-15 iNKT cells with i.p. or i.p. plus i.v. administration against metastatic PSCA^+^ PDCA are effective. The preclinical data presented here suggest the necessity of developing PSCA CAR_sIL-15 iNKT cells clinically for treating late-stage or relapsed PSCA^+^ PDAC patients, either alone or in up-front combination with standard-of-care chemotherapy.

## Methods

### Sex as a biological variable.

Both male and female mice were used in this study.

### Cells.

PSCA^+^ cell lines, Capan-1, MIA PaCa-2, and AsPC-1 cells, and PSCA^–^ cell lines, PANC-1 and BxPC-3, were cultured in DMEM medium containing 10% FBS (Thermo Fisher), penicillin (100 U/ml), and streptomycin (100 μg/ml). All cell lines were verified before use and routinely tested for the absence of mycoplasma using the MycoAlert Plus Mycoplasma Detection Kit (Lonza Bioscience).

### Generation of PSCA CAR_sIL-15 iNKT cells.

For the PSCA CAR vector used for retroviral transduction of primary iNKT cells, the retroviral construct encoded the codon-optimized anti-PSCA single-chain fragment variable (scFv) along with either CD28-CD3ζ-sIL-15-tEGFR domains provided by CytoImmune Therapeutics Inc.

The retrovirus was generated using Lipofectamine 3000 (Thermo Fisher) by transient transfection of GP2-293T cells (Takara) with pRD114-TR plasmid (22). The viral supernatants were collected 48 hours after transfection, filtered through a 0.45 μm filter, aliquoted, and stored at –80°C.

Human PBMCs were obtained from collected circulating blood leukocytes using Ficoll-Paque Plus (GE Healthcare). iNKT cells were isolated from PBMCs using anti-iNKT microbeads (Miltenyi Biotec), and the negative PBMC fraction was irradiated (25 Gy) and aliquoted. Irradiated autologous PBMCs loaded with α-GalCer (100 ng/ml; Kyowa Hakko Kirin) were used to stimulate iNKT cells at a ratio of 1:1. iNKT cells were cultured in RPMI 1640 medium supplemented with 5% human serum (Sigma-Aldrich) and IL-2 (100 IU/mL; NIH). iNKT cells were expanded for 10–12 days and then restimulated with α-GalCer–loaded (100 ng/ml) irradiated autologous PBMCs at a ratio of 2:1. Then, iNKT cells were transduced with retrovirus using Retronectin (Takara) according to the manufacturer’s instructions and further expanded in RPMI 1640 medium supplemented with 5% human serum in the presence of IL-2.

PSCA CAR_sIL-15 iNKT cells were frozen at the concentration of 40 × 10^6^/ml in CS5 using a controlled rate freezer. Frozen cells were thawed rapidly (<1 minute) in a 37°C water bath and cultured in the CellGenix GMP SCGM media (catalog 20802-0500) containing 5% human serum and IL-2.

### Flow cytometry-based assay.

The PDAC cell lines were stained with PE-conjugated mouse anti-human PSCA antibody (clone: 7F5; Santa Cruz Biotechnology) and isotype antibody (BioLegend) to determine the PSCA antigen expression levels. Anti-TCR Vα24-Jα18 (iNKT cell; clone: 6B11), anti-CD3 (clone: OKT3), anti-EGFR (clone: AY13), anti-CD56 (clone: QA17A16), anti-CD8 (clone: SK1), anti-CD4 (clone: A161A1), anti-CD19 (clone: SJ25C1), anti–TIM-3 (clone: F38-2E2), anti–PD-1 (clone: EH12.1), anti-LAG-3 (clone: 7H2C65), and anti-CD45 (clone: HI30) antibodies were all purchased from BioLegend or BD Biosciences. PSCA CAR_sIL-15 iNKT or sIL-15 iNKT cells (4 × 10^5^) were coincubated with an equal amount of tumor cells for 24 hours, and then the activation markers CD69 and CD25 (clone: BC96; BioLegend) using the respective antibodies (clone FN50 and clone BC96, respectively; BioLegend) were detected. The data were acquired with an LSRFortessa X-20 Cell Analyzer (BD Biosciences) and analyzed with FlowJo software, version 10 (Tree Star).

### Apoptosis assay.

Annexin V and 7-AAD (BioLegend) staining was used to examine apoptotic cell death, and PSCA CAR_sIL-15 iNKT or sIL-15 iNKT cells (1 × 10^6^) were harvested and stained according to the manufacturer’s instructions before being subjected to flow cytometry analysis to detect apoptosis. The data were acquired with an LSRFortessa X-20 Cell Analyzer (BD Biosciences) and analyzed with FlowJo software, version 10 (Tree Star).

### Degranulation and intracellular cytokine staining assays.

Degranulation and intracellular cytokine staining assays were performed by coincubating 4 × 10^5^ CAR_sIL-15 iNKT or sIL-15 iNKT cells with 2 × 10^5^ different human PDAC target cells in the presence of 1:50 PE Cyanine7-conjugated CD107a antibody (clone: H4A3; BioLegend). GolgiPlug (BD Biosciences) was added according to the manufacturer’s instructions. After further culture for 4 hours, cells were incubated with antibodies for surface markers and permeabilized for 20 minutes using the Fixation/Permeablization Kit (BD Biosciences), followed by staining with TNF-α and IFN-γ antibodies (clone MAb11 and clone 4S.B3, respectively; BD Biosciences). The data were acquired with an LSRFortessa X-20 Cell Analyzer (BD Biosciences) and analyzed with FlowJo software, version 10.

### Cytolysis assay.

The cytolytic capability of PSCA CAR_sIL-15 iNKT or sIL-15 iNKT cells against PSCA^+^ tumor cells was assessed using an RTCA assay (ACEA Bioscience, xCELLigence RTCA MP) according to the manufacturer’s instructions. Target cells (2 × 10^3^) were seeded on RTCA plates in triplicate and incubated in culture medium at 37°C with 5% CO_2_. After 20−24 hours, PSCA CAR_sIL-15 iNKT/sIL-15 iNKT cells were added to the target cells at the indicated E:T ratios and data were collected at 15-minute intervals. The xCELLigence MP system continuously monitored cell growth for 72 hours.

Capan-1 cells were used as target cells. Primary human NK cells, T cells, or PSCA CAR_sIL-15 iNKT cells were used as effector cells. The target cells were labeled with ^51^Cr for 1 hour. Then the target cells were cocultured with effector cells at different E:T ratios at 37°C for 12 hours. ^51^Cr release was measured with a MicroBeta2 microplate radiometric counter (PerkinElmer). Target cells incubated in complete media or 1% SDS media were used for spontaneous or maximal ^51^Cr release control, respectively. The cell lysis percentages were calculated using the standard formula:


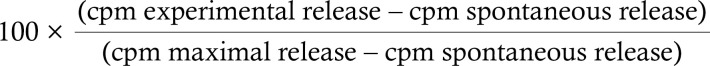


where cpm indicates counts per minute.

### Mouse xenograft models.

For the PDAC metastasis model, male and female 8- to 12-week-old NSG mice (NOD.Cg-Prkdc^scid^ Il2rg^tm1Wjl^/SzJ; Jackson Laboratory) were transplanted with Capan-1 or MIA PaCa-2 PDAC cell lines expressing the fluorescein_ZsGreen gene (Capan-1_luc or MIA PaCa-2_luc) by i.p. injection (2 × 10^5^ cells/mouse). For the PDAC orthotopic model, 40 μL of 2 × 10^5^ MIA PaCa-2_luc tumor cells were surgically injected into the mouse pancreas. For the GR PDAC model, Capan-1 GR_luc and its control Capan-1_luc were i.p. injected (2 × 10^5^ cells/mouse). Prior to any treatment, tumor engraftment was confirmed using BLI imaging. Mice with established tumors were randomly assigned to control or treatment groups and then given cells by i.p. (3 × 10^6^ PSCA CAR_sIL-15 iNKT or sIL-15 iNKT cells) and i.v. injection (i.v.; 1.5 × 10^6^ PSCA CAR_sIL-15 iNKT or sIL-15 iNKT cells). Tumor burden was evaluated using BLI.

For comparing the fresh and frozen CAR iNKT cells, 8- to 12-week-old NSG mice were transplanted with 5 × 10^5^ Capan-1 PDAC cells expressing the fluorescein_ZsGreen gene by i.p. injection on day 1. Prior to treatment, tumor engraftment was confirmed using BLI on day 7. On the same day, mice with established tumors were randomly assigned to 3 groups: (a) saline, (b) freshly produced PSCA CAR_sIL-15 iNKT cells (i.p.: 4 × 10^6^ PSCA CAR_sIL-15 iNKT cells plus i.v.: 2 × 10^6^ PSCA CAR_sIL-15 iNKT cells), and (c) off-the-shelf cryopreserved PSCA CAR_sIL-15 iNKT cells (i.p.: 4 × 10^6^ PSCA CAR_sIL-15 iNKT cells plus i.v.: 2 × 10^6^ PSCA CAR_sIL-15 iNKT cells). Tumor burden was evaluated using BLI.

To detect persistence and kinetics of the infused CAR iNKT cells, the pancreatic tumor model was established by injecting 5 × 10^5^ Capan-1-luc cells on day 1 in NSG mice. On day 7, mice were treated with off-the-shelf cryopreserved PSCA CAR_sIL-15 iNKT cells (i.p.: 4 × 10^6^ PSCA CAR_sIL-15 iNKT cells plus i.v.: 2 × 10^6^ PSCA CAR_sIL-15 iNKT cells per mouse). On days 1, 7, 14, and 21 after the injection of PSCA CAR_sIL-15 iNKT cells, mice were euthanized. Blood, bone marrow, lung, liver, pancreas, kidney, and spleen were harvested and the percentages of iNKT cells among mononuclear cells (hCD45^+^ cells) in each tissue were analyzed by flow cytometry using the anti-human CD45 antibody (clone: 2D1, BioLegend).

To compare the antitumor effects of i.p. plus i.v., i.p. alone, and i.v. alone in vivo, NSG mice were injected with 5 × 10^5^ Capan-1-luc cells on day 1. On day 7, mice were randomly divided into 4 groups: group 1, PBS; group 2, i.p. injection of 4 × 10^6^ PSCA CAR_sIL-15 iNKT cells per mouse; group 3, i.v. injection of 4 × 10^6^ PSCA CAR_sIL-15 iNKT cells per mouse; group 4, i.p. injection of 2 × 10^6^ plus i.v. injection of 2 × 10^6^ of PSCA CAR_sIL-15 iNKT cells per mouse. Tumor burden was evaluated using BLI.

Humanized NSG-SGM3 mice, created by engrafting human PBMCs into the bloodstream of the NSG-SGM3 mice, were injected with 2 × 10^6^ Capan-1-luc cells. Seven days later, mice were treated with off-the-shelf cryopreserved PSCA CAR_sIL-15 iNKT cells (i.p. injection of 4 × 10^6^ plus i.v. injection of 2 × 10^6^ PSCA CAR_sIL-15 iNKT cells per mouse) or PBS control. One day and 3 days after the iNKT cell infusion (defined as days 1 and 3, respectively), mice were euthanized, and sera were harvested for multiplex analysis for CRS-related cytokines using RayBio Human Cytokine Antibody Array 5.

### Study approval.

Experiments and handling of mice were conducted under federal, state, and local guidelines and with approval from the City of Hope Animal Care and Use Committee. To isolate human iNKT cells, T cells or NK cells, peripheral blood cones were collected from healthy donors after written informed consent in the Donor Apheresis Center (DAC) of the City of Hope National Medical Center under institutional review board–approved protocols. Human donor peripheral blood leukocytes from healthy donors were obtained from the City of Hope Michael Amini Transfusion Medicine Center.

### Statistics.

Graphs and data analyses were performed using GraphPad Prism Software, version 8.3.0. Some of these graphs were obtained and modified from Servicer Medical Art. Unless otherwise stated, all data represent at least 3 independent experiments. All data are presented as mean ± SD. Significant differences were analyzed by 2-tailed Student’s *t* test, 1-way ANOVA, 2-way ANOVA, or log-rank test. Significance was defined as *P* < 0.05.

### Data availability.

Values for all data points in graphs are reported in the Supporting Data Values file.

## Author contributions

JY and MAC conceived and designed the project and edited the manuscript. ZD, ZZ, ZL, LT, KYT, and HC conducted experiments. ZD and JZ performed data analyses. LM provided samples ZD, LT, LSW, and JY wrote the manuscript. JY and MAC acquired funding. All authors discussed the results and commented on the manuscript.

## Supplementary Material

Supplemental data

Supporting data values

## Version 1 04/15/2025

Electronic publication

## References

[1] Islami F (2022). American Cancer Society’s report on the status of cancer disparities in the United States, 2021. CA Cancer J Clin.

[2] Rahib L (2014). Projecting cancer incidence and deaths to 2030: the unexpected burden of thyroid, liver, and pancreas cancers in the United States. Cancer Res.

[3] Binenbaum Y (2015). Gemcitabine resistance in pancreatic ductal adenocarcinoma. Drug Resist Updat.

[4] Lee HS, Park SW (2016). Systemic chemotherapy in advanced pancreatic cancer. Gut Liver.

[5] Maalej KM (2023). CAR-cell therapy in the era of solid tumor treatment: current challenges and emerging therapeutic advances. Mol Cancer.

[6] Sterner RC, Sterner RM (2021). CAR-T cell therapy: current limitations and potential strategies. Blood Cancer J.

[7] Dotti G (2009). Fifteen years of gene therapy based on chimeric antigen receptors: “are we nearly there yet?”. Hum Gene Ther.

[8] June C (2012). T-cell therapy at the threshold. Nat Biotechnol.

[9] Liu L (2020). Enhanced CAR-T activity against established tumors by polarizing human T cells to secrete interleukin-9. Nat Commun.

[10] Heczey A (2014). Invariant NKT cells with chimeric antigen receptor provide a novel platform for safe and effective cancer immunotherapy. Blood.

[11] Ulanova M (2000). Antigen-specific regulation of CD1 expression in humans. J Clin Immunol.

[12] Metelitsa LS (2004). Natural killer T cells infiltrate neuroblastomas expressing the chemokine CCL2. J Exp Med.

[13] Tachibana T (2005). Increased intratumor Valpha24-positive natural killer T cells: a prognostic factor for primary colorectal carcinomas. Clin Cancer Res.

[14] Morris ES (2005). NKT cell-dependent leukemia eradication following stem cell mobilization with potent G-CSF analogs. J Clin Invest.

[15] Pillai AB (2007). Host NKT cells can prevent graft-versus-host disease and permit graft antitumor activity after bone marrow transplantation. J Immunol.

[16] Casorati G (2012). Invariant natural killer T cells reconstitution and the control of leukemia relapse in pediatric haploidentical hematopoietic stem cell transplantation. Oncoimmunology.

[17] Zhou X (2024). CAR-redirected natural killer T cells demonstrate superior antitumor activity to CAR-T cells through multimodal CD1d-dependent mechanisms. Nat Cancer.

[18] Gu Z (2000). Prostate stem cell antigen (PSCA) expression increases with high gleason score, advanced stage and bone metastasis in prostate cancer. Oncogene.

[19] Saeki N (2010). Prostate stem cell antigen: a Jekyll and Hyde molecule?. Clin Cancer Res.

[20] Li E (2017). PSCA promotes prostate cancer proliferation and cell-cycle progression by up-regulating c-Myc. Prostate.

[21] Wente MN (2005). Prostate stem cell antigen is a putative target for immunotherapy in pancreatic cancer. Pancreas.

[22] Teng KY (2022). Off-the-shelf prostate stem cell antigen-directed chimeric antigen receptor natural killer cell therapy to treat pancreatic cancer. Gastroenterology.

[23] Liu D (2012). IL-15 protects NKT cells from inhibition by tumor-associated macrophages and enhances antimetastatic activity. J Clin Invest.

[24] Lellouche L (2021). Systemic therapy in metastatic pancreatic adenocarcinoma: current practice and perspectives. Ther Adv Med Oncol.

[25] Jia Y (2019). The role of GLI-SOX2 signaling axis for gemcitabine resistance in pancreatic cancer. Oncogene.

[26] Samulitis BK (2015). Gemcitabine resistant pancreatic cancer cell lines acquire an invasive phenotype with collateral hypersensitivity to histone deacetylase inhibitors. Cancer Biol Ther.

[27] Lai HY (2012). Cytokine profiles in various graft-versus-host disease target organs following hematopoietic stem cell transplantation. Cell Transplant.

[28] Shah Z (2024). Human anti-PSCA CAR macrophages possess potent antitumor activity against pancreatic cancer. Cell Stem Cell.

[29] An B (2018). CD1d is a novel cell-surface marker for human monocytic myeloid-derived suppressor cells with T cell suppression activity in peripheral blood after allogeneic hematopoietic stem cell transplantation. Biochem Biophys Res Commun.

[30] Sharma V (2022). CCR4^+^ monocytic myeloid-derived suppressor cells are associated with the increased epithelial-mesenchymal transition in pancreatic adenocarcinoma patients. Immunobiology.

[31] Gabitass RF (2011). Elevated myeloid-derived suppressor cells in pancreatic, esophageal and gastric cancer are an independent prognostic factor and are associated with significant elevation of the Th2 cytokine interleukin-13. Cancer Immunol Immunother.

[32] Xu JY (2019). The importance of a conjoint analysis of tumor-associated macrophages and immune checkpoints in pancreatic cancer. Pancreas.

[33] Shi B (2021). The scavenger receptor MARCO expressed by tumor-associated macrophages are highly associated with poor pancreatic cancer prognosis. Front Oncol.

[34] De Santo C (2008). Invariant NKT cells reduce the immunosuppressive activity of influenza A virus-induced myeloid-derived suppressor cells in mice and humans. J Clin Invest.

[35] Cortesi F (2018). Bimodal CD40/Fas-dependent crosstalk between iNKT cells and tumor-associated macrophages impairs prostate cancer progression. Cell Rep.

[36] Takami M (2024). Anti-Vα24Jα18 TCR antibody tunes iNKT cell responses to target and kill CD1d-negative tumors in an Fcγ RII (CD32)-dependent manner. Cancer Res Commun.

[37] Delfanti G (2022). Adoptive immunotherapy with engineered iNKT cells to target cancer cells and the suppressive microenvironment. Front Med (Lausanne).

[38] Janakiram NB (2017). Loss of natural killer T cells promotes pancreatic cancer in LSL-Kras^G12D/+^ mice. Immunology.

[39] Gorini F (2017). Invariant NKT cells contribute to chronic lymphocytic leukemia surveillance and prognosis. Blood.

[40] Lee JM (2012). The restoration of myeloid-derived suppressor cells as functional antigen-presenting cells by NKT cell help and all-trans-retinoic acid treatment. Int J Cancer.

[41] Ko HJ (2009). Immunosuppressive myeloid-derived suppressor cells can be converted into immunogenic APCs with the help of activated NKT cells: an alternative cell-based antitumor vaccine. J Immunol.

[42] Delfanti G (2022). TCR-engineered iNKT cells induce robust antitumor response by dual targeting cancer and suppressive myeloid cells. Sci Immunol.

[43] Bian J, Almhanna K (2021). Pancreatic cancer and immune checkpoint inhibitors-still a long way to go. Transl Gastroenterol Hepatol.

[44] Yu P (2012). Simultaneous inhibition of two regulatory T-cell subsets enhanced Interleukin-15 efficacy in a prostate tumor model. Proc Natl Acad Sci U S A.

