# Supplementary material for: Off-the-shelf invariant NKT cells expressing anti-PSCA CAR and IL-15 promote pancreatic cancer regression in mice
Source: J Clin Invest. 2025 Apr 15;135(8):e179014. doi: 10.1172/JCI179014 (PMC11996875; doi:10.1172/JCI179014)
Supplement: Supplemental data [file jci-135-179014-s055.pdf]

Supplementary Information

Off-the-shelf invariant NKT cells expressing anti-PSCA CAR and IL-15 promote pancreatic cancer regression in mice

Zhenyu Dai[#1], Zheng Zhu[#1], Zhiyao Li[#1], Lei Tian[#2,3], Kun-Yu Teng[1], Hanyu Chen[1], Li-Shu Wang[1], Jianying Zhang[4], Laleh Melstrom[5], Michael A Caligiuri[1,6,7] *, Jianhua Yu[2,3] *

Affiliations:

[1]Department of Hematology and Hematopoietic Cell Transplantation, City of Hope National Medical Center, Los Angeles, California 91010, USA.

[2]Division of Hematology/Oncology, Department of Medicine, School of Medicine, University of California, Irvine, California CA 92697, USA

[3]The Clemons Family Center for Transformative Cancer Research, Chao Family Comprehensive Cancer Center, University of California, Irvine, California CA 92697, USA

[4]Department of Computational and Quantitative Medicine, City of Hope National Medical Center, Los Angeles, California 91010, USA.

[5]Division of Surgical Oncology, Department of Surgery, City of Hope National Medical Center, Los Angeles, California 91010, USA.

[6]Hematologic Malignancies Research Institute, City of Hope National Medical Center, Los Angeles, California 91010, USA.

[7]City of Hope Comprehensive Cancer Center, Los Angeles, California 91010, USA.

# These authors equally contributed to this work.

*Corresponding authors: Jianhua Yu, PhD, jianhuay@uci.edu; Michael A Caligiuri, MD, mcaligiuri@coh.org

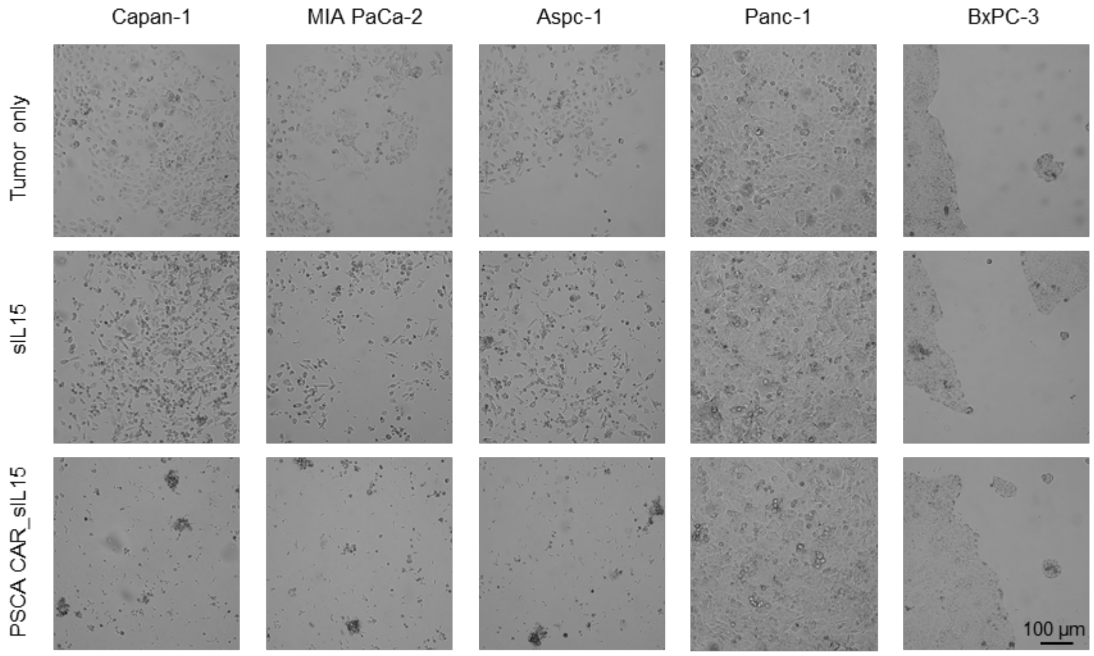

Figure S1. After 90 hours of co-incubation, original images show killing of target cells by PSCA CAR sIL15/sIL15 iNKT cells. PSCA CAR_sIL15 iNKT cells demonstrated robust killing activity against PSCA^{+} tumor cell lines, including Capan-1 cells, MIA Paca-2 cells, and Aspc-1 cells, in contrast to sIL15 iNKT cells. However, neither sIL15 iNKT cells nor PSCA CAR sIL15 iNKT cells exhibited cytotoxicity against the PSCA^{-} cell lines, Panc-1 and BxPC-3. The experiment was repeated with 3 donors.

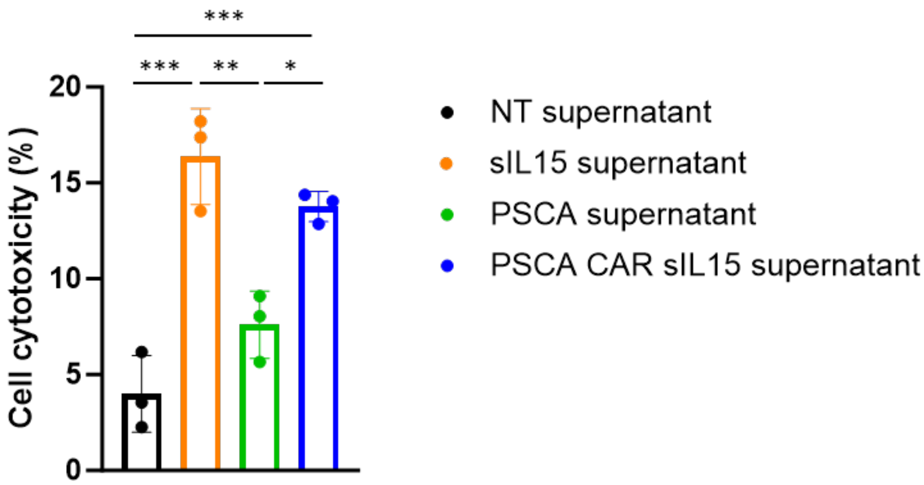

Figure S2. Freshly isolated human primary T cells were cultured in the presence of supernatants from non-transduced iNKT (NT supernatant), sIL15 iNKT, PSCA CAR iNKT, or PSCA CAR_sIL15 iNKT cells for two days. Capan-1 cells were labeled with ^{51}Cr and served as target cells. The labeled target cells were added to the cultured T cells in the presence of respective supernatants for additional 12 hours. The cytotoxicity levels were measured by ^{51}Cr release assay. n = 3 donors. NT vs. PSCA, P = 0.1629; NT vs. sIL15, P = 0.0002; PSCA vs. PSCA CAR sIL15, P = 0.0158; PSCA vs. sIL15, P = 0.0019; sIL15 vs. PSCA CAR sIL15, P = 0.3799. Statistical analyses were performed by one-way ANOVA with P values corrected for multiple comparisons by the Bonferroni method.

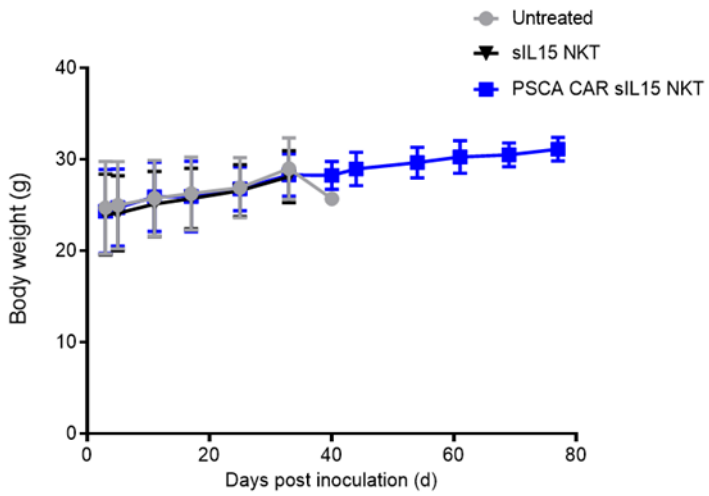

Figure S3. Mouse body weight monitoring after treatment with PSCA CAR_sIL15 iNKT cells in a human metastatic pancreatic cancer model established by injection of Capan-1_luc cells into NSG mice.

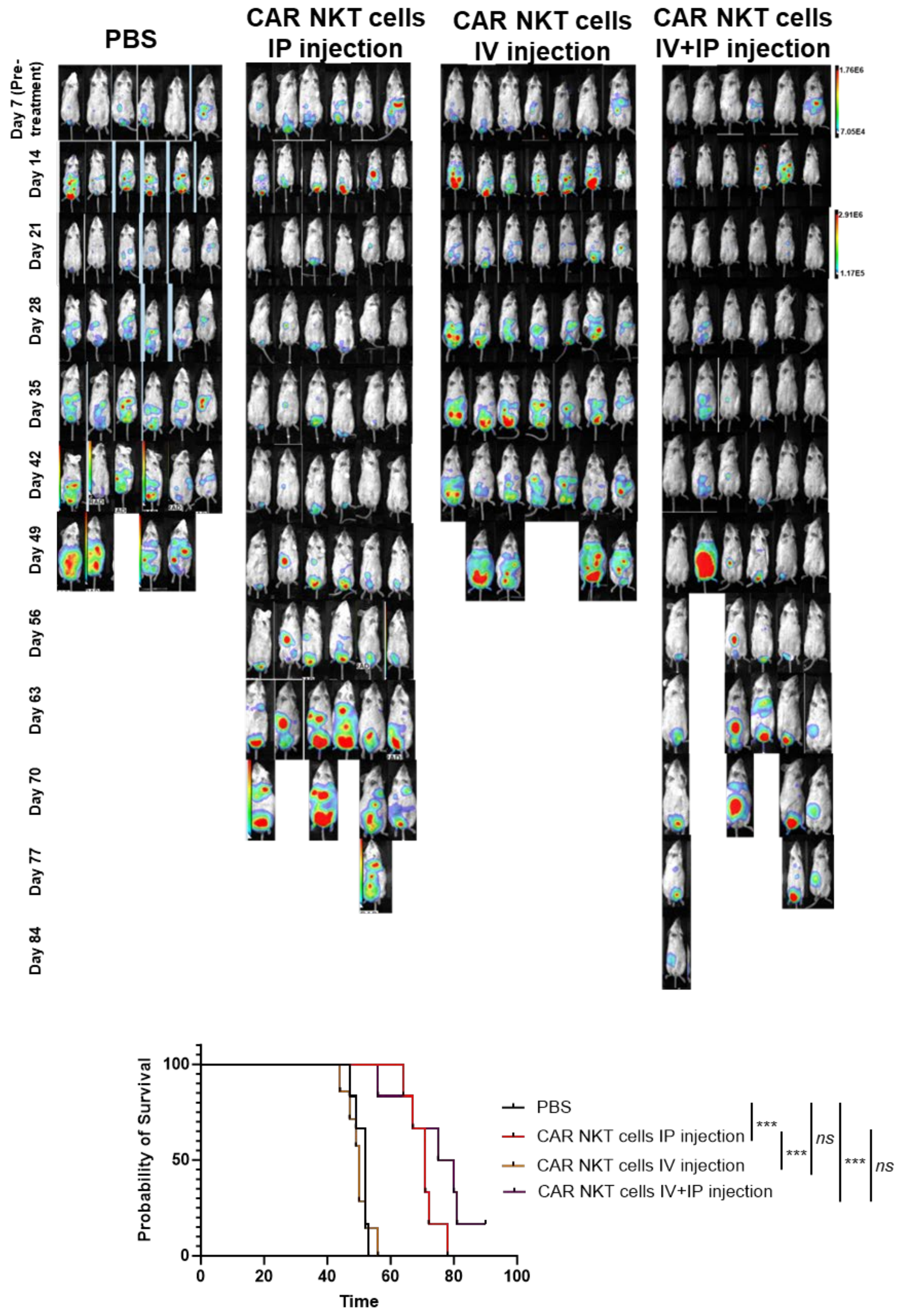

Figure S4. NSG mice were injected with 5×10^5 Capan-1-luc cells on day 1. On day 7, mice were randomly divided into 4 groups. Group 1: PBS. Group 2: CAR iNKT cells, IP 4×10^6 per mouse. Group 3: CAR iNKT cells, IV 4×10^6 per mouse. Group 4: CAR iNKT cells, IP 2×10^6 plus IV 2×10^6 per mouse. Tumor growth was monitored by bioluminescence. Overall survival was estimated by the Kaplan Meier method (n = 6 or 7/group).

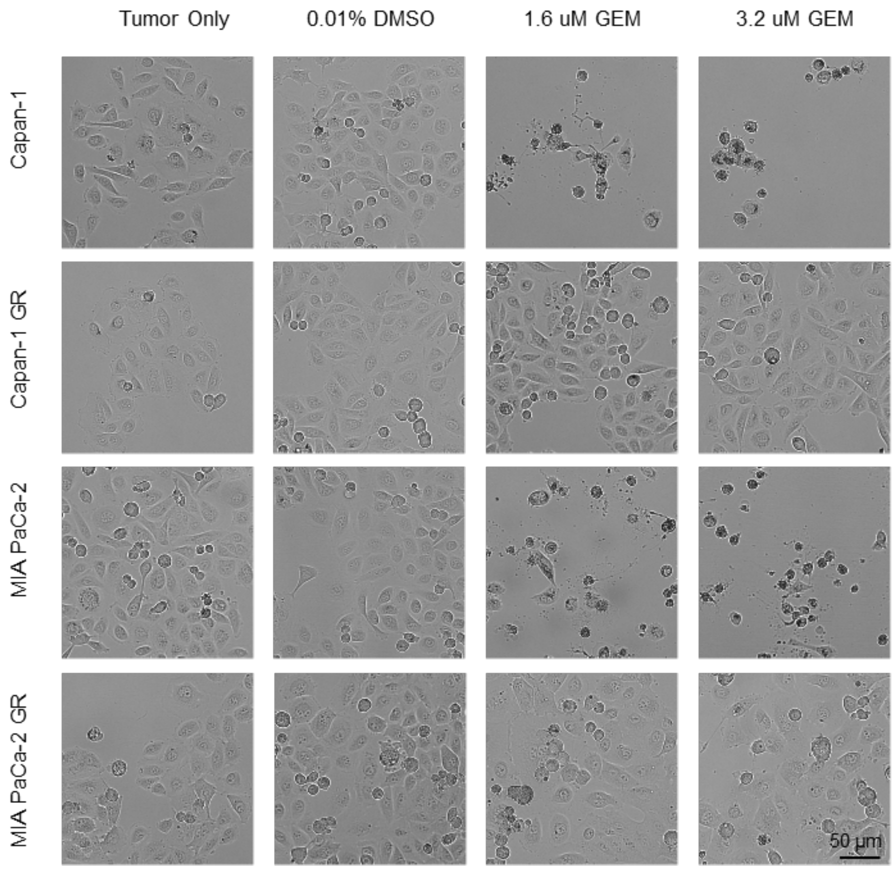

Figure S5. Representative images of gemcitabine-resistant cell lines (Capan-1 GR and MIA Paca-2 GR) and parental cell lines after co-culture with different concentrations of gemcitabine and control (0.01% DMSO) for 72 hours. The experiment was repeated three times.

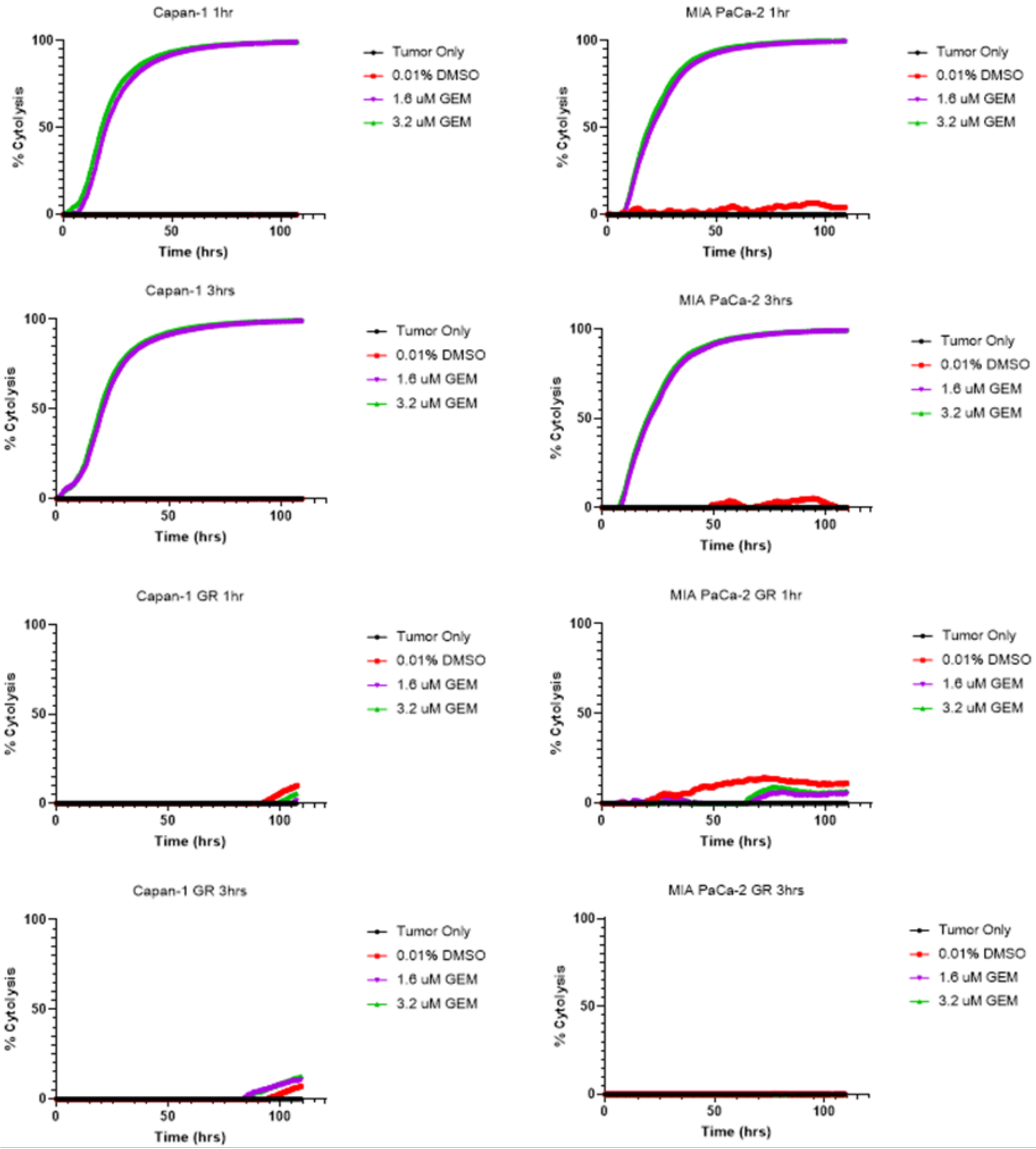

Figure S6. RTCA analysis of the effect of different concentrations of gemcitabine on gemcitabine-resistant cell lines (Capan-1 GR and MIA Paca-2 GR), compared with their respective parental cell lines. All cells were pre-treated with the indicated concentrations of gemcitabine for 1 hour or 3 hours, after which the culture medium was replaced with fresh media without gemcitabine for RTCA analysis. The experiment was repeated three times.

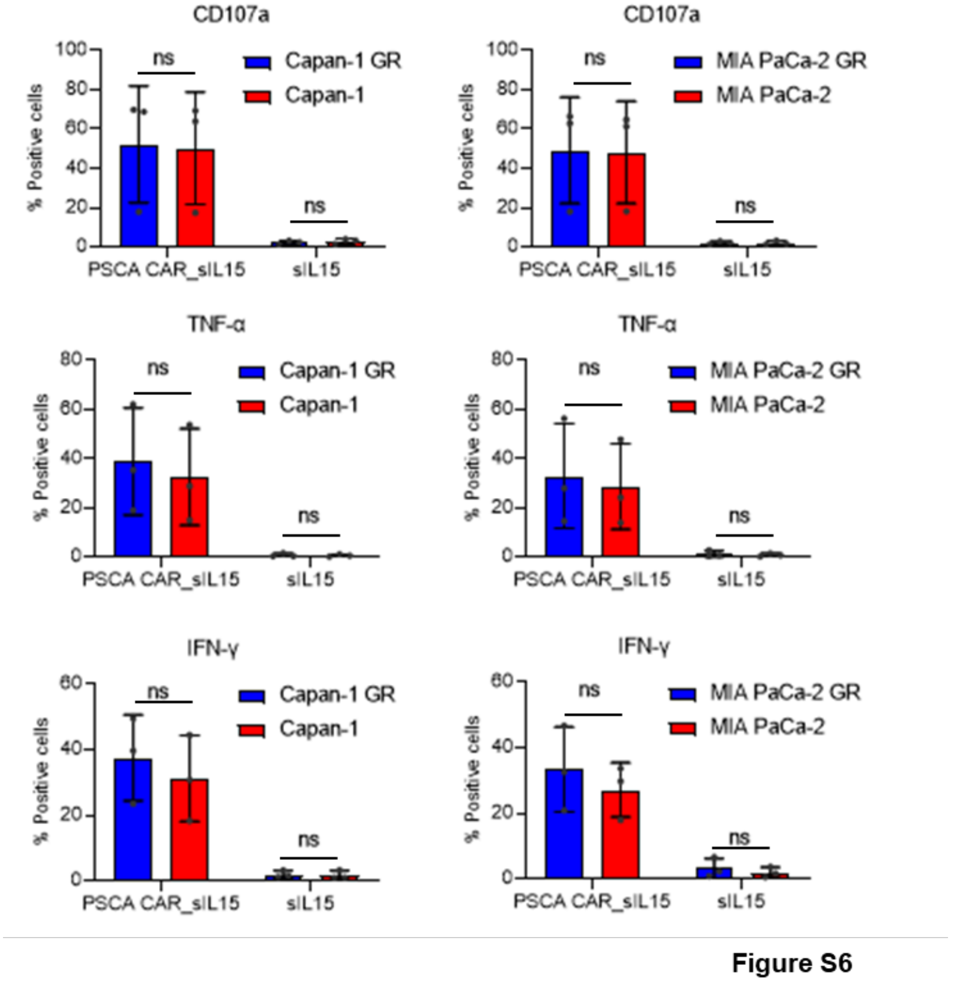

Figure S7. Expression of CD107a (a surrogate marker for degranulation) and intracellular cytokines TNF-α and IFN-γ (gated on iNKT cells) was detected in PSCA CAR_sIL15/sIL15 iNKT cells co-cultured with Capan-1 GR, Capan-1 MIA Paca-2 GR or MIA Paca-2 for 24 hours, assessed by flow cytometry. Data represent ± SD (n = 3).

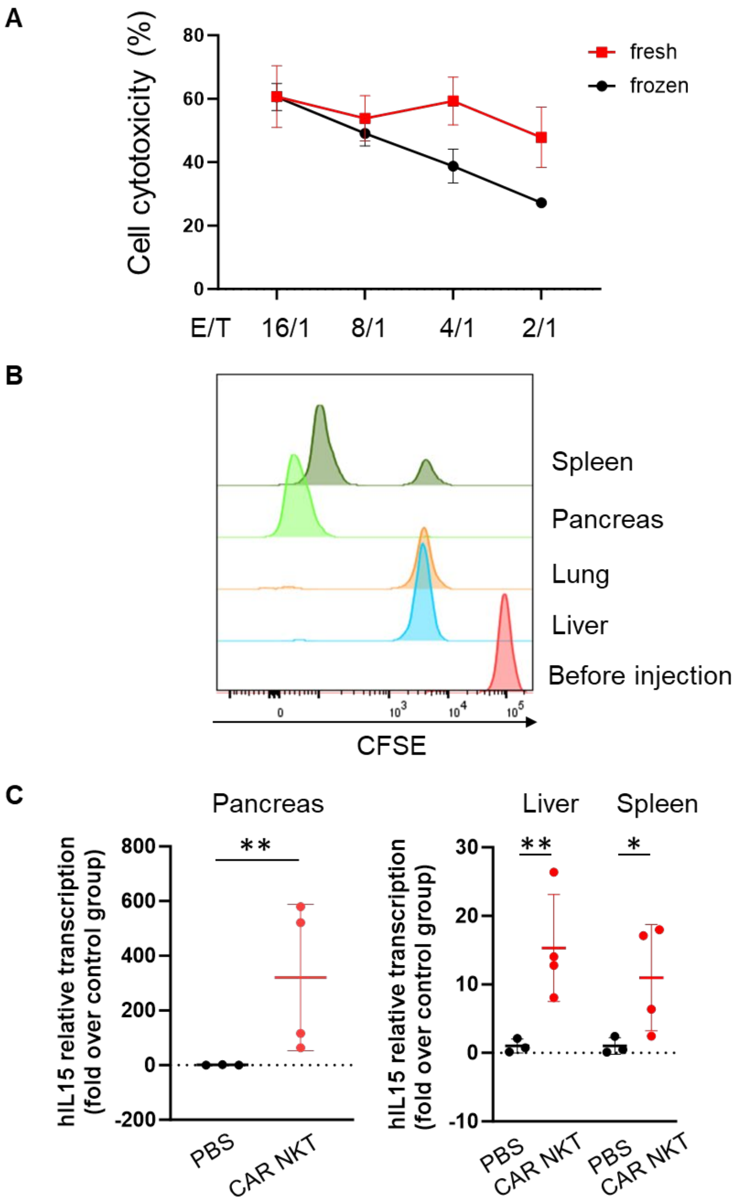

Figure S8. (A) The cytotoxicity levels of freshly produced CAR iNKT cells and "off-the-shelf" cryopreserved (frozen) CAR iNKT cells were measured by a standard 51Cr release assay against Capan-1 target cells at the indicated effector:target ratios. Fresh vs. frozen: P = 0.021, two-sided t-test. n = 3 donors. **(B)** NSG mice were injected with 5×10^5 Capan-1-luc cells on day 1. On day 7, the mice were injected with CFSE-labeled iNKT cells (IP: 4×10^6, IV: 2×10^6 per mouse). The lungs, liver, pancreas, and spleen were harvested two days after the iNKT cell injection. iNKT cell proliferation was detected by flow cytometry to quantify $hCD45^+$ CFSE. The results were repeated with 4 mice, yielding similar results. **(C)** NSG mice were injected IP with 5×10^5 Capan-1-luc cells on day 1. On day 7, the mice were injected with frozen PSCA CAR_sIL15 iNKT cells (IP: 4×10^6, IV: 2×10^6 per mouse). PBS was injected as a control. The liver, pancreas, and spleen were harvested 4 days after the injection of PSCA CAR_sIL15 iNKT cells. The expression levels of IL-15 were assessed by RT-qPCR. n = 3 or 4 mice/group. Statistical analyses were conducted using a two-sided t-test with log-rank adjustment.

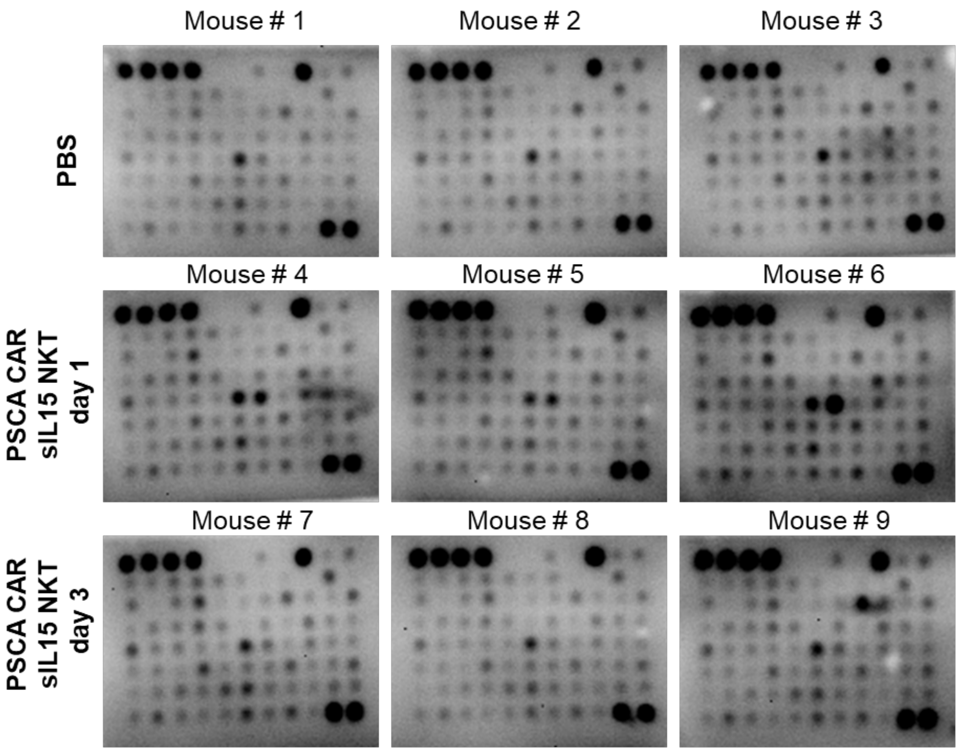

Map

Pos	Pos	Pos	Pos	Neg	Neg	ENA-78	G-CSF	GM-CSF	GRO	GRO-alpha
I-309	IL-1alpha	IL-1beta	IL-2	IL-3	IL-4	IL-5	IL-6	IL-7	IL-8	IL-10
IL12-p40	IL-13	IL-15	IFN-gamma	MCP-1	MCP-2	MCP-3	M-CSF	MDC	MIG	MIP-1 beta
MIP-1-delta	RANTES	SCF	SDF-1	TARC	TGF-beta 1	TNF-alpha	TNF-beta	EGF	IGF-1	Angiogenin
Oncostatin M	TPO	VEGF	PDGF-BB	Leptin	BDNF	BLC	CK beta 8-1	Eotaxin	Eotaxin-2	Eotaxin-3
FGF-4	FGF-6	FGF-7	FGF-9	Flt-3 Ligand	Fractalkine	GCP-2	GDNF	HGF	IGFBP-1	IGFBP-2
IGFBP-3	IGFBP-4	IL-16	IP-10	LIF	LIGHT	MCP-4	MIF	MIP-3-alpha	NAP-2	NT-3
NT-4	Osteopontin	Osteoprotegerin	PARC	PIGF	TGF- b 2	TGF- b 3	TIMP-1	TIMP-2	Pos	Pos

Figure S9. Secretome analysis of sera collected from PBS- or PSCA CAR_sIL15 iNKT-treated humanized mice bearing pancreatic tumors at the time of euthanasia. Secretome images of three mice in each group and the map displaying the location to detect individual cytokines are shown.
